# Preparation of Macrometallocycle and Selective Sensor for Copper Ion

**DOI:** 10.1038/s41598-018-29356-z

**Published:** 2018-07-19

**Authors:** Yingjie Liu, Zhixiang Zhao, Qingxiang Liu

**Affiliations:** 1 Tianjin Key Laboratory of Process Measurement and Control, Institute of Robotics and Autonomous Systems, Tianjin University, Tianjin, 300072 China; 2 Key Laboratory of Inorganic-Organic Hybrid Functional Materials Chemistry (Tianjin Normal University), Ministry of Education; Tianjin Key Laboratory of Structure and Performance for Functional Molecules, College of Chemistry, Tianjin Normal University, Tianjin, 300387 China

## Abstract

Two bis-imidazolium salts 1,8-bis[2’-(*N*-R-imidazoliumyl)acetylamino]naphthalene chloride (L^1^H_4_·Cl_2_: R = Et; L^2^H_4_·Cl_2_: R = ^n^Bu), as well as their four NHC metal complexes [L^1^H_2_Ag]Cl (1), [L^1^Ni] (2), [L^2^Ni] (3) and [L^1^H_2_Hg(HgCl_4_)] (4) have been synthesized. In each of the cationic moieties of complexes 1 or 4, there is a groove-like 14-membered macrometallocycle, and each macrometallocycle is consisted of one biscarbene ligand L^1^H_2_ and one metal ion (silver(I) ion for 1 and mercury(II) ion for 4). Three 6-membered cycles are contained in each molecule of complexes 2 or 3. Additionally, the selective recognition of macrometallocycle 1 for Cu^2+^ was studied with the methods of fluorescence and ultraviolet spectroscopy, ^1^H NMR titrations, MS and IR spectra. The experimental results display macrometallocycle 1 can discriminate Cu^2+^ from other cations effectively.

## Introduction

The detection of Cu^2+^ occupies an important position in host-guest chemistry because it plays a crucial part in chemistry, biology and environmental science^1–3^. As a trace element in the body, copper are key components of hemocyanin and some enzymes. Ingesting excess or deficient Cu^2+^ will cause serious illness, such as Alzheimer’s and Wilson’s diseases, haematological manifestations and liver damage^4–12^. Excess Cu^2+^ can also destroy the aquatic ecosystem, and disturb the nutrient absorption and transport of some plants^13^. Among the detection of Cu^2+^, the fluorescent chemosensor is one of significant tools due to its high sensitivity and the simplicity of equipment^14–16^. So far, a variety of types of fluorescent chemosensors for Cu^2+^ have been reported, such as organic small molecules and MOFs^17–23^. Besides, Liu and co-workers reported a sensor based on porous conjugated polymers for Cu^2+^, and it is high sensitivity and selectivity^24^. Though some chemosensors for Cu^2+^ have appeared, the design and synthesis of new practical chemosensors are still desirable.

In the process of searching for suitable chemosensors for Cu^2+^, we focused on N-heterocyclic carbene (NHC) metal complexes because of their diverse structures, such as macrocycle^25–29^, molecular rectangle^30–32^ and groove^33,34^. In a large number of complexes, cyclic NHC metal complexes have favorable recognition capability for metal ions^35–39^, because this kind of host can capture effectively metal ions through several kinds of forces (electrostatic force, M···M interactions, M···X interactions and M···π interactions). Herein, we report the synthesis of bis-imidazolium salts 1,8-bis[2′-(*N*-R-imidazoliumyl)acetylamino]naphthalene chloride (**L**^**1**^**H**_**4**_**·Cl**_**2**_: R = Et; **L**^**2**^**H**_**4**_**·Cl**_**2**_: R = ^n^Bu), as well as the preparation and structure of four NHC complexes [L^1^H_2_Ag]Cl (**1**), [L^1^Ni] (**2**), [L^2^Ni] (**3**) and [L^1^H_2_Hg(HgCl_4_)] (**4**). Additionally, we studied the selective recognition of macrometallocycle **1** for Cu^2+^ with the methods of fluorescence and ultraviolet spectroscopy, ^1^H NMR titrations, MS and IR spectra.

## Results and Discussion

### Synthesis and characterization of L^1^H_4_·Cl_2_ and L^2^H_4_·Cl_2_

**Figure 1:**
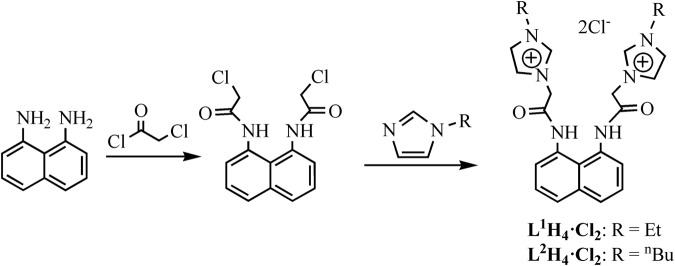
Preparation of Precursors **L**^**1**^**H**_**4**_**·Cl**_**2**_ and **L**^**2**^**H**_**4**_**·Cl**_**2**_.

As shown in Fig. 1, 1,8-diaminonaphthalene reacted with chloroacetyl chloride to give 1,8-di(2′-chloroacetylamino)naphthalene, which further reacted with *N*-R-imidazole (R = Et or ^n^Bu) to generate bis-imidazolium salts **L**^**1**^**H**_**4**_**·Cl**_**2**_ and **L**^**2**^**H**_**4**_**·Cl**_**2**_. Precursors **L**^**1**^**H**_**4**_**·Cl**_**2**_ and **L**^**2**^**H**_**4**_**·Cl**_**2**_ remain stable in the air, and can be dissolved in DMSO, dichloromethane and acetonitrile, but their solubility is poor in benzene, diethyl ether and petroleum ether. In the ^1^H NMR spectra of **L**^**1**^**H**_**4**_**·Cl**_**2**_ and **L**^**2**^**H**_**4**_**·Cl**_**2**_, the proton signals (NC*H*N) of imidazolium appear at *δ* = 9.47 and 9.50 ppm, and these values are analogous to those of known imidazolium compounds^33,40–46^.

### Synthesis and general characterization of complexes 1–4

**Figure 2:**
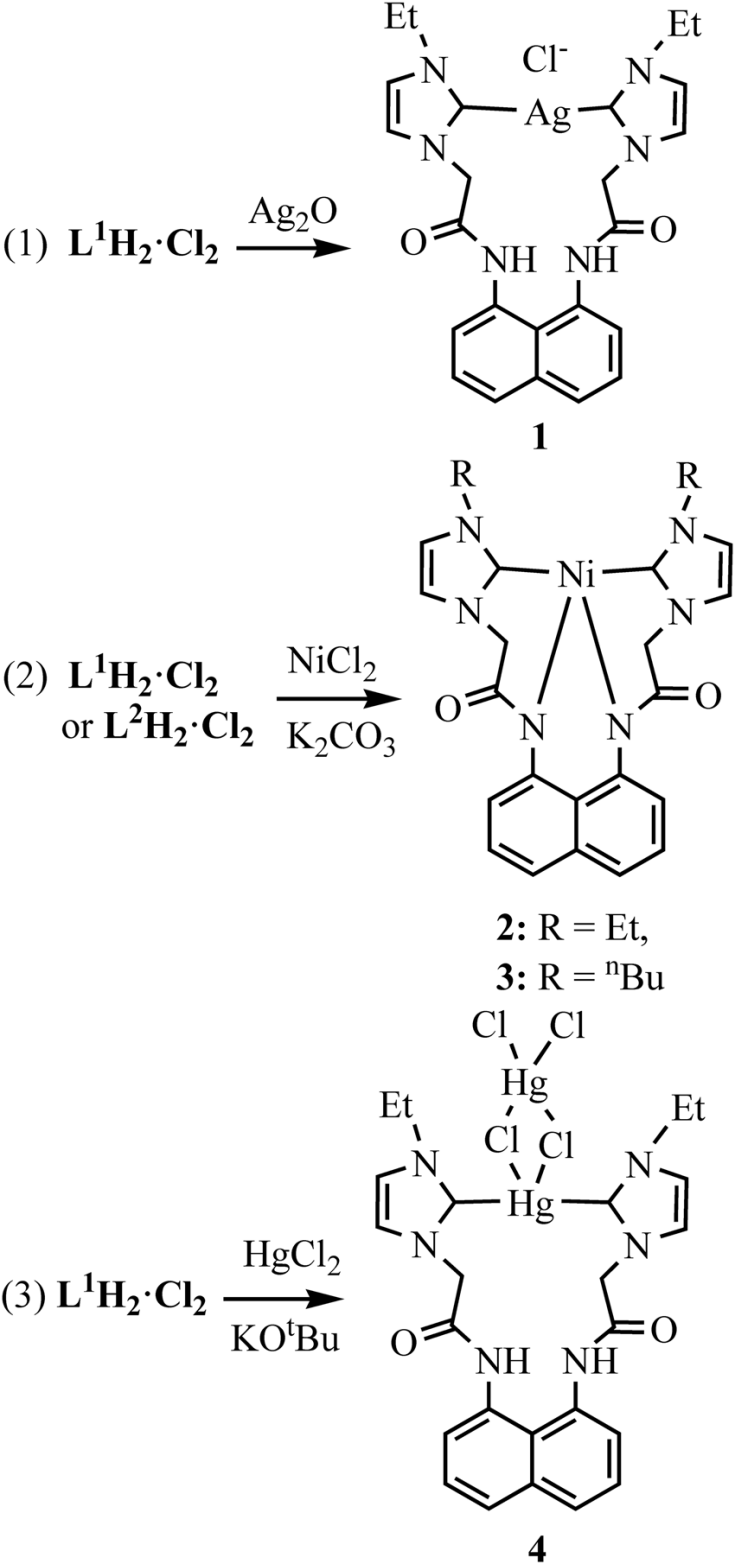
Preparation of Complexes **1–4**.

The synthesis of NHC silver(I) complex [L^1^H_2_Ag]Cl (**1**) was accomplished via the reaction of **L**^**1**^**H**_**4**_**·Cl**_**2**_ with Ag_2_O in CH_3_CN/DMSO (Fig. 2). The reactions of **L**^**1**^**H**_**4**_**·Cl**_**2**_ or **L**^**2**^**H**_**4**_**·Cl**_**2**_ with NiCl_2_ in the presence of K_2_CO_3_ in CH_3_CN/DMSO afforded NHC nickel(II) complexes [L^1^Ni] (**2**) and [L^2^Ni] (**3**). The reaction of **L**^**1**^**H**_**4**_**·Cl**_**2**_ with HgCl_2_ in the presence of KO^t^Bu in CH_3_CN/DMSO gave NHC mercury(II) complex [L^1^H_2_Hg(HgCl_4_)] (**4**).

The crystals of complexes **1–4** were obtained via slow adding Et_2_O to their solutions. Complexes **1–4** can be dissolved in DMSO and CH_3_CN, but they are scarce soluble in benzene, diethyl ether and petroleum ether. The solution of complex **1** is slightly light-sensitive. The proton signals (NC*H*N) of imidazolium disappear in the ^1^H NMR spectra of **1–4** due to the introduction of metals, and other proton signals are analogous to **L**^**1**^**H**_**4**_**·Cl**_**2**_ or **L**^**2**^**H**_**4**_**·Cl**_**2**_. In the ^13^C NMR spectra of **1**, no carbene carbon signal is found, and this phenomenon may be the fluxional behavior of the NHC silver(I) complexes^47–49^. The carbene carbon signals of **2–4** are observed at 175.0–176.8 ppm, which are consistent with other NHC metal complexes in literatures^50–60^.

### Structure of complexes 1–4

**Figure 3:**
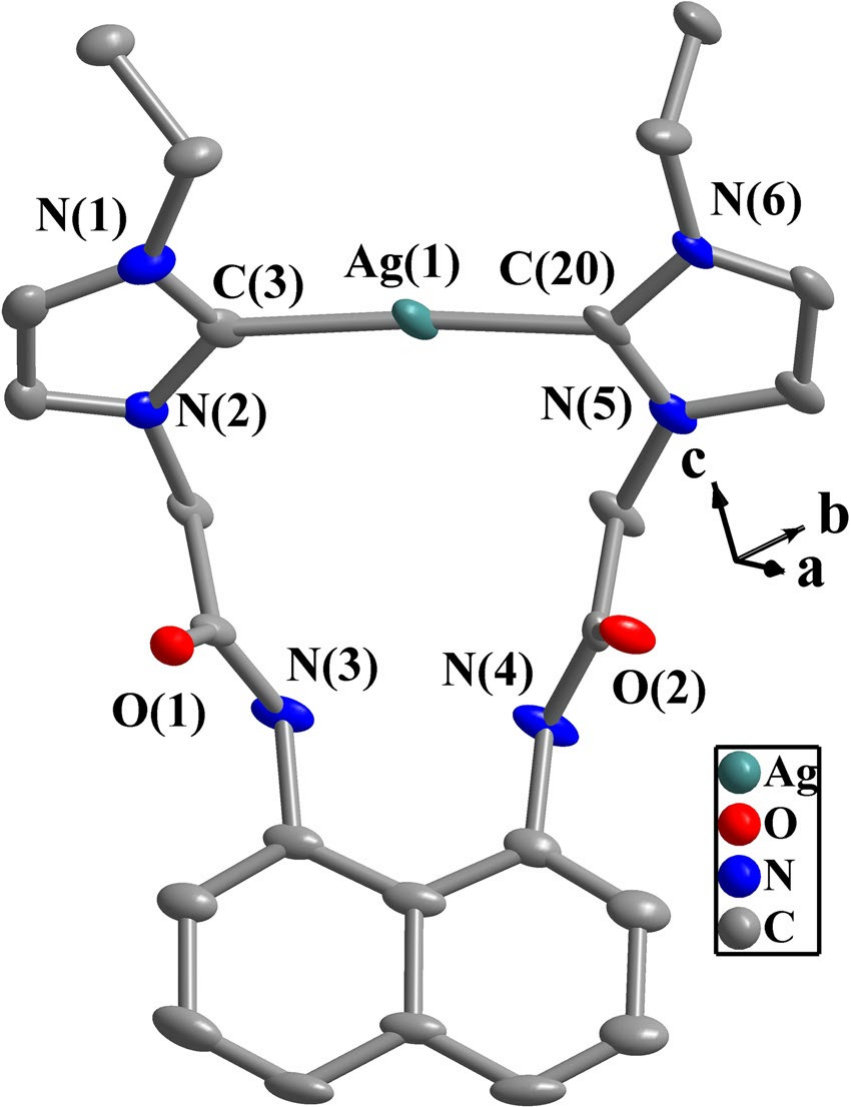
Perspective view of **1** and anisotropic displacement parameters depicting 50% probability. Selected bond lengths (Å) and angles (°): Ag(1)-C(3) 2.100(8), Ag(1)-C(20) 2.074(8); C(3)-Ag(1)-C(20) 175.3(3), N(1)-C(3)-N(2) 104.0(7), N(5)-C(20)-N(6) 104.5(6).

**Figure 4:**
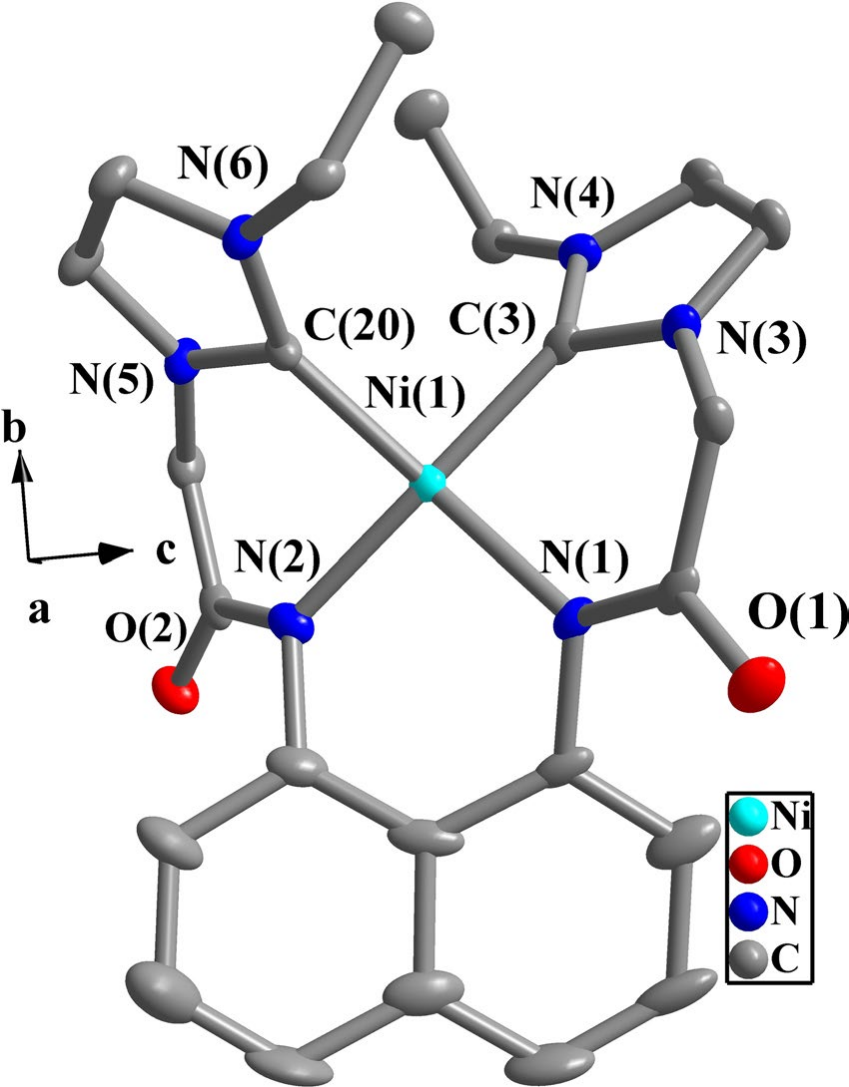
Perspective view of **2** and anisotropic displacement parameters depicting 50% probability. Selected bond lengths (Å) and angles (°):C(3)-Ni(1) 1.858(5), C(20)-Ni(1) 1.864(5), N(1)-Ni(1) 1.933(4), N(2)-Ni(1) 1.925(4); C(3)-Ni(1)-C(20) 91.2(2), N(1)-Ni(1)-N(2) 94.6(2), N(3)-C(3)-N(4) 104.9(4), C(3)-Ni(1)-N(1) 89.7(1), C(20)-Ni(1)-N(2) 88.1(2).

**Figure 5:**
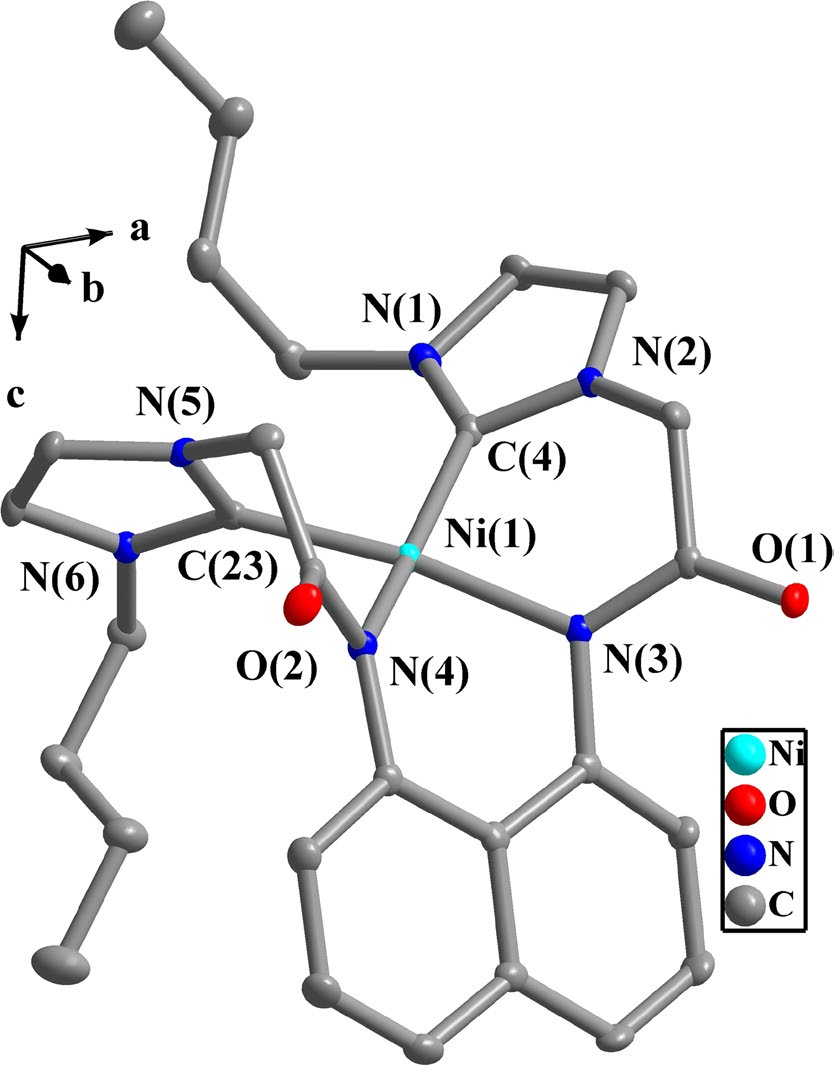
Perspective view of **3** and anisotropic displacement parameters depicting 50% probability. Selected bond lengths (Å) and angles (°): N(3)-Ni(1) 1.918(1), C(4)-Ni(1) 1.900(2), C(23)-Ni(1) 1.871(2), N(4)-Ni(1) 1.929(1); C(23)-Ni(1)-C(4) 97.9(1), N(3)-Ni(1)-N(4) 87.0(8), N(1)-C(4)-N(2) 103.5(1), N(5)-C(23)-N(6) 104.9(1), C(4)-Ni(1)-N(3) 91.8(9), C(23)-Ni(1)-N(4) 84.4(9).

**Figure 6:**
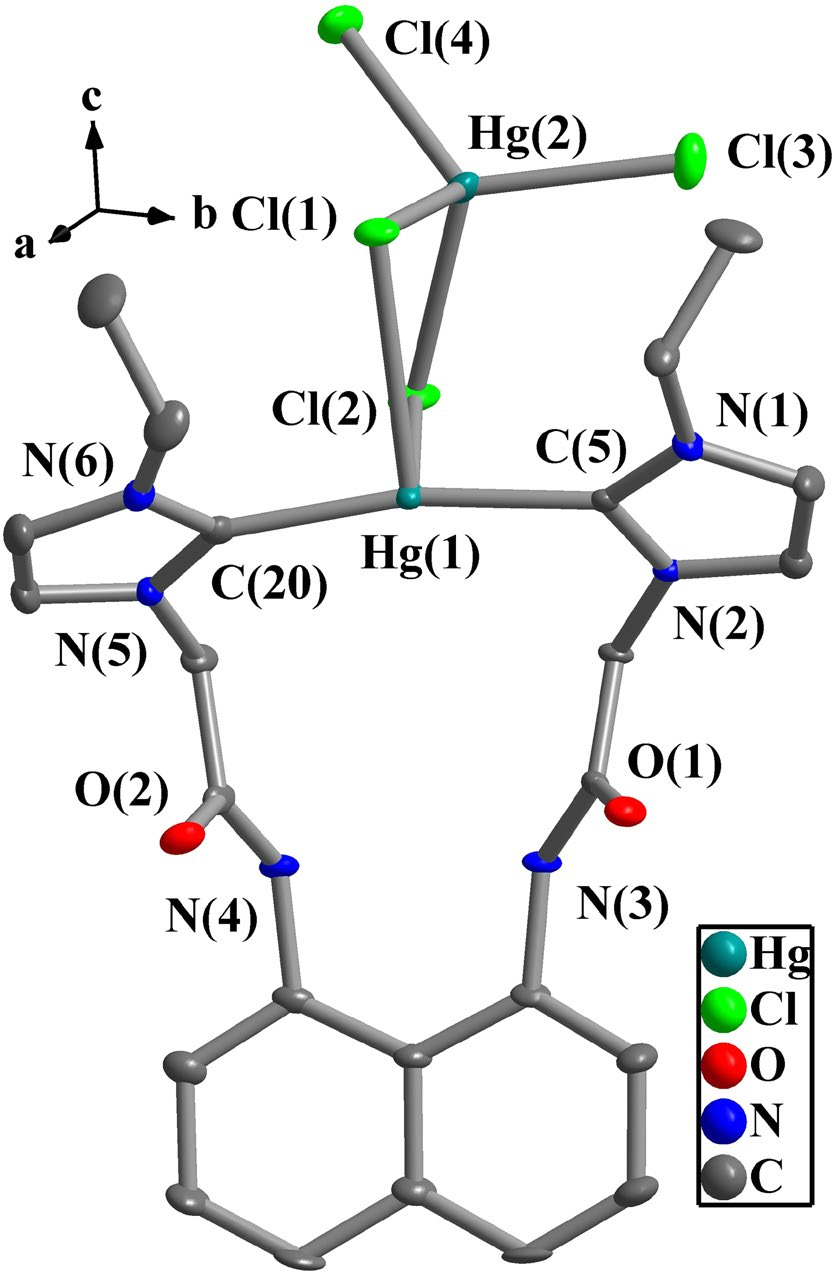
Perspective view of **4** and anisotropic displacement parameters depicting 50% probability. Selected bond lengths (Å) and angles (°): Hg(1)-C(5) 2.073(6), Hg(1)-C(20) 2.081(7), Hg(1)-Cl(1) 2.880(1), Hg(2)-Cl(1) 2.514(1), Hg(2)-Cl(2) 2.557(1), Hg(2)-Cl(3) 2.437(1), Hg(2)-Cl(4) 2.418(1); C(5)-Hg(1)-C(20) 168.6(2), N(1)-C(5)-N(2) 106.3(5), N(5)-C(20)-N(6) 106.2(6).

In complexes **1**–**4** (Figs 3–6), the N-C-N angles are between 103.5(1)° and 106.3(5)°, and these values are consistent with those of literatures^47–49,61^. One 14-membered macrometallocycle is contained in each of the molecules of complexes **1** or **4**. By contrast, three 6-membered cycles in each molecule of **2** or **3** are observed. In the same ligand for **1**–**4**, the naphthalene plane and two imidazole planes form the dihedral angles of 51.5(5)–75.9(8)° (Table S1 in Supporting Information). Two imidazole planes in the same NHC-metal-NHC unit form the dihedral angles of 9.6(5)–14.2(4)° for **1** and **4**. In complexes **2** and **3**, the dihedral angles formed by two imidazole planes are in the range of 74.9(1)–83.4(3)°.

In complex **1**, the arrangement of C(3)-Ag(1)-C(20) is almost linear with the angle of 175.3(3)°, and the distances of Ag(1)-C(3) and Ag(1)-C(20) are 2.074(8) Å and 2.100(8) Å. Both are comparable with those of known NHC Ag(I) complexes^47–49^.

In complexes **2** or **3**, two acetylamino groups (-CON*H-*) and two imdazolium moieties of precursors **L**^**1**^**H**_**4**_**·Cl**_**2**_ or **L**^**2**^**H**_**4**_**·Cl**_**2**_ are deprotonated in the presence of K_2_CO_3_. As a result, Ni(II) ion is coordinated to two carbene atoms and two nitrogen atoms to adopt a quadrilateral geometry with slight distortion. The bond distances of C-Ni and N-Ni are 1.858(5)–1.900(2) Å and 1.918(1)–1.933(4) Å, respectively. The bond angles of C-Ni-C, N-Ni-N and C-Ni-N are 91.2(2)–97.9(1)°, 87.0(8)−94.6(2)° and 84.4(9)–169.9(9)°, respectively. Similar values were also reported in other literatures about NHC Ni(II) complexes^61^.

Both of Hg(1) and Hg(2) in complex **4** are tetra-coordinated. The distances of Hg(1)-C(5) and Hg(1)-C(20) are 2.073(6) Å and 2.081(7) Å, and the bond angle of C(5)-Hg(1)-C(20) is 168.6(2)°. The distances of Hg(2)-Cl (2.418(2)–2.557(1) Å) are shorter than that of Hg(1)-Cl(1) (2.880(1) Å). A distorted Hg_2_Cl_2_ quadrangular arrangement is formed by Hg(1), Cl(1), Hg(2) and Cl(2), in which the dihedral angle between the Cl(1)-Hg(1)-Cl(2) plane and the Cl(1)-Hg(2)-Cl(2) plane is 30.5(8)°. The Hg···Hg separation of 3.815(5) Å suggests the nonexistence of metal-metal interactions between both Hg(II) ions (van der Waals Radii of mercury = 1.70 Å)^62,63^.

### Recognition of Cu^2+^ using 1 as a chemosensor

The screening experiments of complexes **1**–**4** for some cations (Li^+^, Na^+^, K^+^, NH_4_^+^, Ag^+^, Ca^2+^, Co^2+^, Ni^2+^, Cu^2+^, Zn^2+^, Cd^2+^, Cr^3+^, Al^3+^, Pb^2+^ and Hg^2+^, and their anions are NO_3_^−^) via fluorescence spectroscopy in CH_3_CN at 25 °C were carried out. The fluorescence intensities of complexes **2**–**4** didn’t change after adding cations. However, the fluorescence emission of complex **1** decreased remarkably after adding Cu^2+^, and other cations did not have similar phenomenon. Therefore, complex **1** was selected as a chemosensor to process recognition investigation of cations.

**Figure 7:**
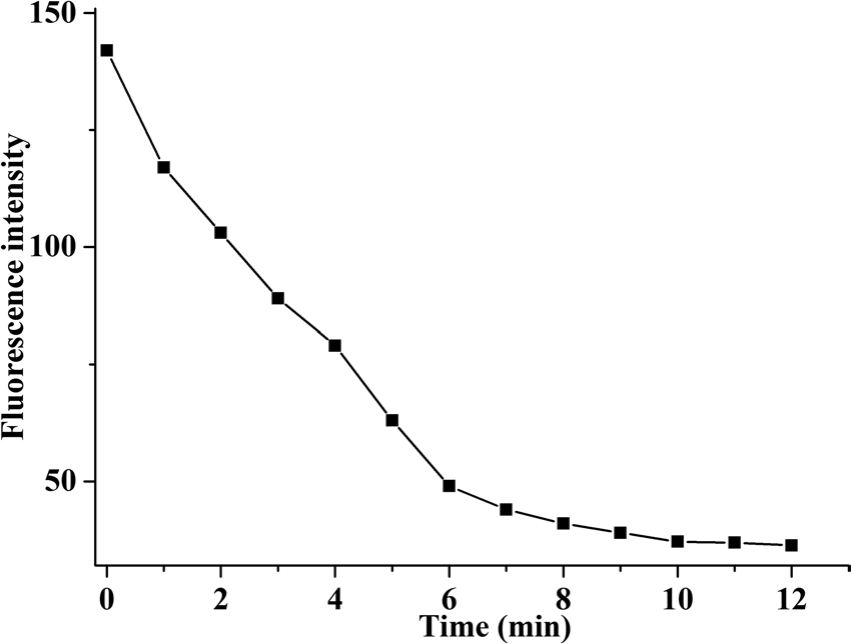
Plot of fluorescence intensity of **1** (2.0 × 10^−6^ mol/L) and Cu^2+^ (20 × 10^−6^ mol/L) as a function of time in minutes.

To evaluate the response time of complex **1** to Cu^2+^, the time-dependent plot was measured (Fig. 7). The results showed that the interactions between Cu^2+^ and **1** can cause fluorescence quenching, in which fluorescence intensity quickly reduced within 6 minutes, and then the tendency slowed down. The fluorescence quantum yields (Φ) of **L**^**1**^**H**_**4**_**·Cl**_**2**_ and complex **1** using 1-aminonaphthalene as fluorescence standard (Φ = 0.39) were measured^64^. The fluorescence quantum yields of **L**^**1**^**H**_**4**_**·Cl**_**2**_ and complex **1** were determined to be 0.16 and 0.21, and the latter was higher than the former. It may be originated to the incorporation of metal-ligand coordination interactions^65,66^.

**Figure 8:**
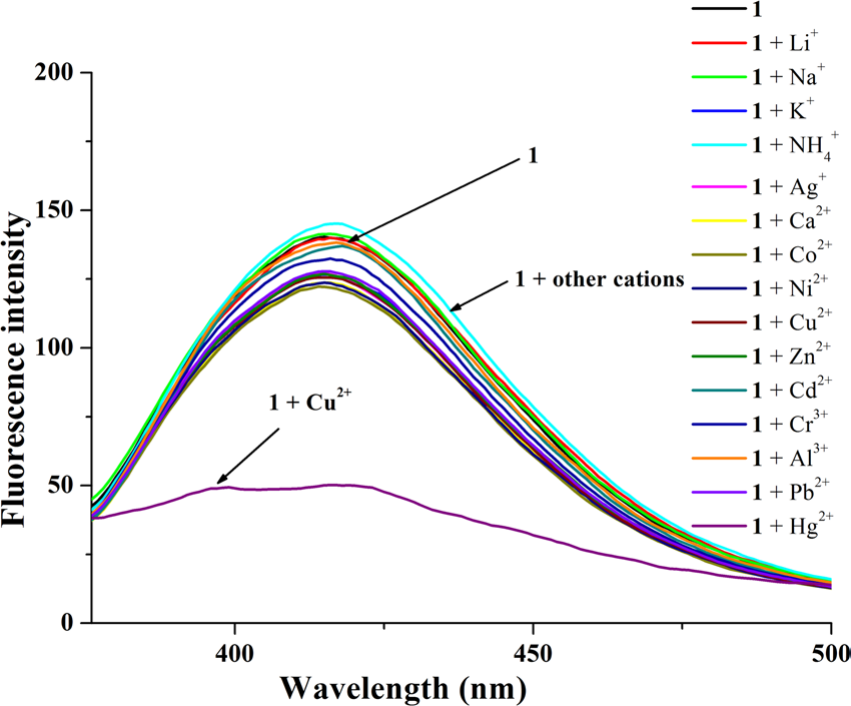
Fluorescence spectra of **1** (2.0 × 10^−6^ mol/L) and 10 equiv. of some cations (Li^+^, Na^+^, K^+^, NH_4_^+^, Ag^+^, Ca^2+^, Co^2+^, Ni^2+^, Cu^2+^, Zn^2+^, Cd^2+^, Cr^3+^, Al^3+^, Pb^2+^ and Hg^2+^) in CH_3_CN at 25 °C.

As shown in Fig. 8, complex **1** showed a fluorescence emission band at *ca*. 415 nm, which originated from conjugated bis(acetylamino)-naphthalene (*λ*_ex_ = 330 nm). When 10 equiv. of Li^+^, Na^+^, K^+^, NH_4_^+^, Ag^+^, Ca^2+^, Co^2+^, Ni^2+^, Zn^2+^, Cd^2+^, Cr^3+^, Al^3+^, Pb^2+^ and Hg^2+^ were added, the fluorescence intensity of **1** had no observable change. However, the significant fluorescence quenching of **1** was observed after adding 10 equiv. of Cu^2+^. In UV/vis experiment, upon addition of Cu^2+^ to the solution of **1**, the absorption of **1** at *ca*. 250–350 nm increased remarkably, but other cations had no similar influence on the absorption of **1** (Fig. S1 in the Supporting Information). The experiment results showed that **1** can discriminate Cu^2+^ from other cations effectively.

**Figure 9:**
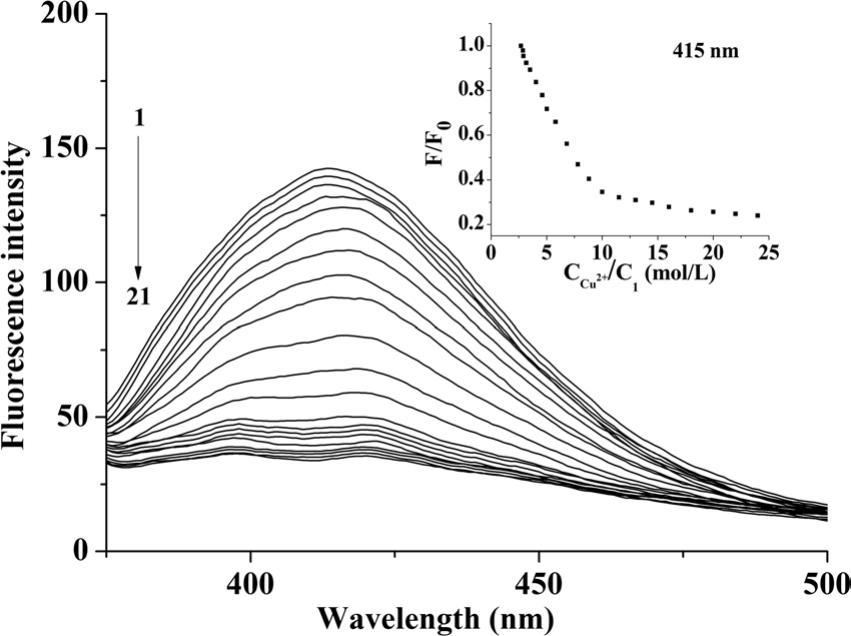
Fluorescence titration spectra of **1** (2.0 × 10^−6^ mol/L) in the presence of different concentrations of Cu^2+^ in CH_3_CN at 25 °C. C_Cu_^2+^ for curves 1–21 (from top to bottom) are 0, 0.6, 1.2, 1.8, 2.6, 3.4, 4.2, 5.0, 5.8, 6.8, 7.8, 8.8, 10.0, 11.5, 13.0, 14.5, 16.0, 18.0, 20.0, 22.0, 24.0 × 10^−6^ mol/L (*λ*_ex_ = 330 nm). Inset: variation of fluorescence quenching *F*/*F*_0_ of **1** with increasing Cu^2+^ concentration.

In the fluorescence titration experiments (Fig. 9), upon the titration of Cu^2+^ into solutions of **1** in CH_3_CN at 25 °C, the fluorescence intensities of **1** at *ca*. 415 nm decreased gradually. In the inset of Fig. 9, the fluorescence intensities of **1** went down quickly in the ratios of C_Cu_^2+^/C_**1**_ being 0 to 10:1. When the ratio ascended to 20:1, the quenching rate slowed down. Finally, fluorescence intensities remained unchanged even though more Cu^2+^ was added. The quenching behaviors of Cu^2+^ on the fluorescence of **1** were found to follow a conventional Stern-Volmer relationship^67,68^ (equation (1)).

1 $${F}_{0}/F=1+{K}_{SV}{{C}_{Cu}}^{2+}$$

where *F*_0_ and *F* are the fluorescence intensities of **1** in the absence and presence of Cu^2+^, and C_Cu_^2+^ is the concentration of Cu^2+^. The equation reveals that *F*_0_/*F* increases in direct proportion to the increasing concentration of Cu^2+^, and the Stern-Volmer constant *K*_*SV*_ defines the quenching efficiency of Cu^2+^.

**Figure 10:**
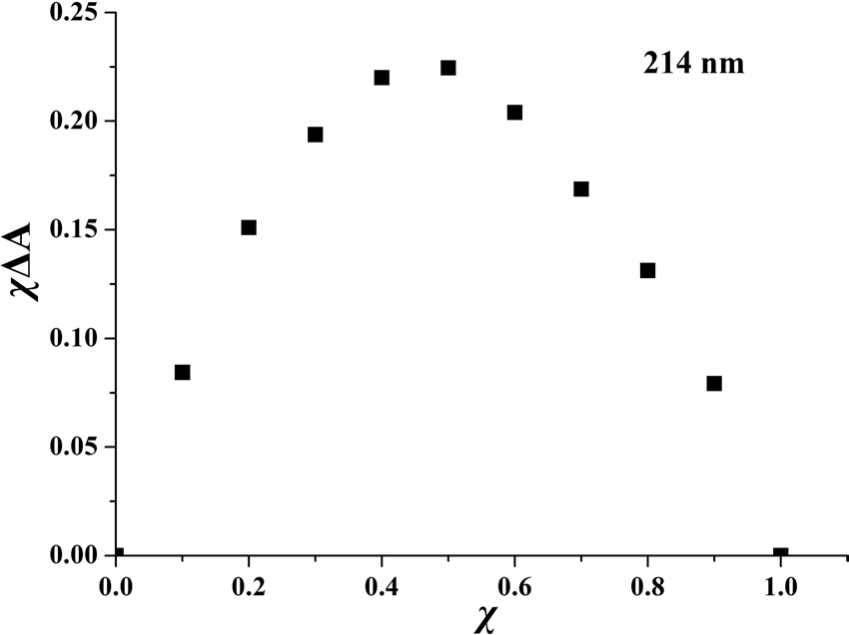
The Job’s plot of **1** toward Cu^2+^ at 214 nm. *χ* is the molar fraction of **1**. It illustrates the host-guest fluorescence quenching occurs in 1:1 complexation.

The *K*_*SV*_ value for **1**·Cu^2+^ was calculated as 5.68 × 10^5^ M^−1^ (R = 0.999) by using the equation (1) (Fig. S2). As shown in Fig. S3, the detection limit was estimated to be 1.5 × 10^−7^ mol/L^34^. To furthur comfirm the complexation stoichiometry between **1** and Cu^2+^, a Job’s plot analysis at 214 nm was carried out (Fig. 10)^62,63^. The *χΔA* values for **1**·Cu^2+^ reached a maximum when molar fractions (*χ*) of **1** was 0.5, and it indicated stoichiometric ratio was 1:1. Where total concentration was a constant, and *ΔA* was the discrepancy of the absorption bands.

To test the ability to resist interference of other cations, the competition experiments were conducted (Fig. S4), where **1** (2.0 × 10^−6^ mol/L) was mixed with 5 equiv. of Li^+^, Na^+^, K^+^, NH_4_^+^, Ag^+^, Ca^2+^, Co^2+^, Ni^2+^, Zn^2+^, Cd^2+^, Cr^3+^, Al^3+^, Pb^2+^ or Hg^2+^, and then 5 equiv. of Cu^2+^ was added. The presence of other cations did not cause any significant changes in the emission of **1**·Cu^2+^.

Analogous to Fig. 8, the decrease of fluorescence intensities of **1** were also observed after the addition of other copper(II) salts (1.0 × 10^−5^ mol/L) with different counter anions (Br^−^, SO_4_^2−^, OAc^−^, Cl^−^, NO_3_^−^ and CO_3_^2−^) (Fig. S5). Thus, the different anions did not obviously influence on the binding between **1** and Cu^2+^. Reversible binding of **1** with Cu^2+^ was also carried out (Fig. S6). The addition of 10 equiv. of EDTA to a mixture of **1** (2.0 × 10^−6^ mol/L) and Cu^2+^ (20 × 10^−6^ mol/L) resulted in the increase of fluorescence intensity at 415 nm, and the fluorescence intensity was approximately equal to that of **1**, which signified the regeneration of the free **1**. The fluorescence intensity decreased upon the addition of Cu^2+^ again. This result showed that **1** was a good chemosensor for Cu^2+^ with admirable reversibility and regeneration capacity.

### Interactions of 1 with Cu^2+^

**Figure 11:**
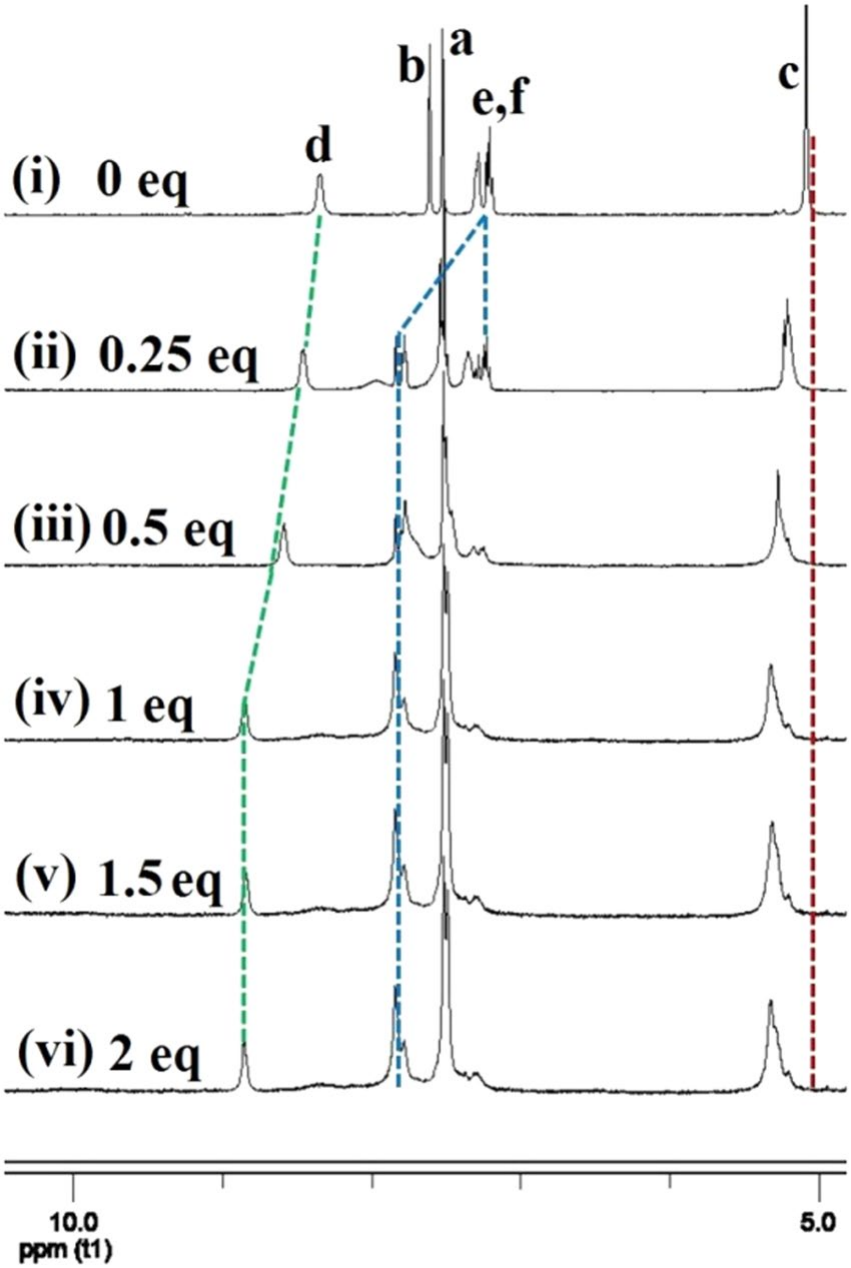
Partial ^1^H NMR spectra in DMSO-*d*_6_. (**i**) **1**; (**ii**) **1** and 0.25 equiv. of Cu^2+^; (**iii**) **1** and 0.5 equiv. of Cu^2+^; (**vi**) **1** and 1 equiv. of Cu^2+^; (**v**) **1** and 1.5 equiv. of Cu^2+^; (**vi**) **1** and 2 equiv. of Cu^2+^.

**Figure 12:**
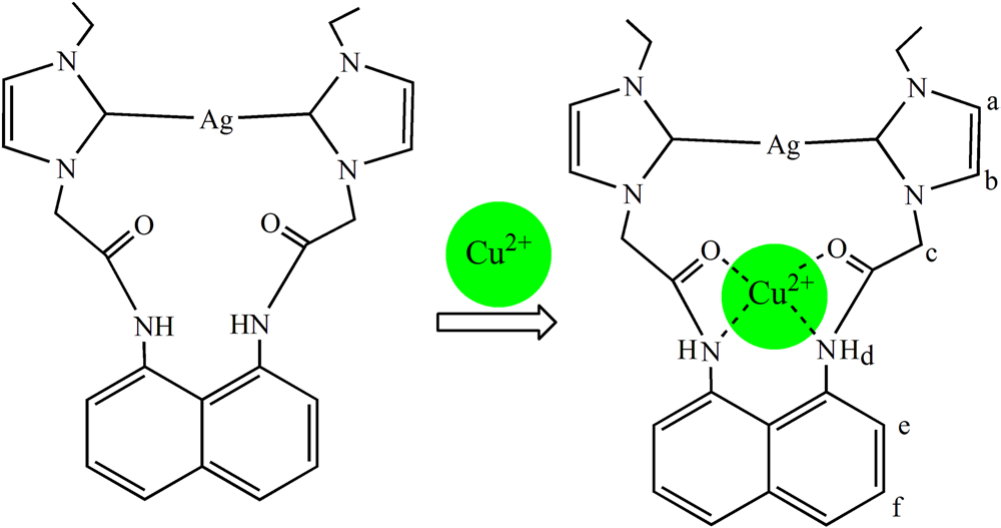
The interactions of **1** with Cu^2+^.

The potential binding sites of **1** for Cu^2+^ may be oxygen atoms, nitrogen atoms and π systems (including O···Cu^2+^ interactions, N···Cu^2+^ interactions and π···Cu^2+^ interactions). To get detailed information on how **1** bound with Cu^2+^, we studied the data of ^1^H NMR titrations (C_Cu_^2+^/C_**1**_ was from 0 to 2.0 equiv.) in DMSO-*d*_6_ (Fig. 11). Upon the addition of 1 equiv. of Cu^2+^, the proton signal on NH (*H*d) had a large downfield shift by 0.92 ppm (Fig. 11(iv)), and the proton signals of *H*e and *H*f on naphthalene ring also shifted to downfield (*ca*. 0.27 ppm), which may be attributed to electron-withdrawing effect of Cu^2+^ due to Cu^2+^···N interactions (Fig. 12). The proton signal of *H*c on CH_2_ attached to C=O shifted to downfield (*ca*. 0.25 ppm), which may be attributed to electron-withdrawing effect of Cu^2+^ due to Cu^2+^···O interactions. More equivalents of Cu^2+^ did not cause further change of chemical shifts of *H*c-*H*f (Fig. 11(v,vi)), which showed the combination ratio between **1** and Cu^2+^ was 1:1.

Additional evidence for the combination ratio between **1** and Cu^2+^ was obtained through high-resolution mass spectra of **1**·Cu^2+^ (Fig. S7). The observation of m/z (318.3) for (**1**·Cu^2+^)/2 furthur comfirmed the formation of a 1:1 complex. This finding agreed with the result of Job’s plot (Fig. 10). The IR spectra of **1** and **1**·Cu^2+^ were measured for more information about how **1** bound with Cu^2+^. In Fig. S8, we found that several absoption bands have changed after adding Cu^2+^. The *υ*_(C=O)_ varied from 1660 cm^−1^ to 1683 cm^−1^, *υ*_(N-H)_ varied from 3378 cm^−1^ to 3382 cm^−1^, and *δ*_(N-H)_ varied from 1617 cm^−1^ to 1629 cm^−1^, respectively.

By analyzing the structure of **1** and above experiment results, we can conclude that **1** bound with Cu^2+^ mainly through Cu^2+^···O and Cu^2+^···N interactions. Once complex **1**·Cu^2+^ was formed, the photo-induced electron transfer (PET) process from the imidazole rings to naphthalene ring was switched on and it led to the quench of fluorescence emission of **1**^69,70^. We tried to cultivate the single crystal of **1**·Cu^2+^, but unsuccessful.

## Conclusion

In conclusion, we prepared and characterized two bis-imidazolium salts **L**^**1**^**H**_**2**_**·Cl**_**2**_ and **L**^**2**^**H**_**2**_**·Cl**_**2**_, as well as their four NHC metal complexes **1**–**4**. In each molecule of **1** or **4**, one 14-membered groove-like macrometallocycle was contained. Additionally, the selective recognition of macrometallocycle **1** for Cu^2+^ was studied with the methods of fluorescence and ultraviolet spectroscopy, ^1^H NMR titrations, MS and IR spectra. The experimental results displayed macrometallocycle **1** can distinguish Cu^2+^ from other cations effectively. *K*_*SV*_ value of 5.68 × 10^5^ M^−1^ for **1**·Cu^2+^ based on a 1:1 association equation analysis was obtained through fluorescence titrations. The detection limit was calculated as 1.5 × 10^−7^ mol/L, which indicated that **1** is sensitive for Cu^2+^. In literatures, some peptide sensors for Cu^2+^ were reported^71–76^, and their association constants and detection limits were in the ranges of 10^4^–10^6^ M^−1^ and 10^−5^–10^−7^ mol/L. Compared with these sensors, sensor **1** showed similar binding ability and good sensitivity to Cu^2+^. Further investigation for new NHC metal complexes from **L**^**1**^**H**_**2**_**·Cl**_**2**_, **L**^**2**^**H**_**2**_**·Cl**_**2**_ and similar to precursors are still under way.

## Experimental Section

### General procedures

*N*-ethyl-imidazole and *N*-^n^butyl-imidazole were prepared according to the methods of literature reported^67,77^. Schlenk techniques were used in all manipulations. All the reagents for synthesis and analyses were of analytical grade and used without further purification. Melting points were determined with a Boetius Block apparatus. ^1^H and ^13^C NMR spectra were recorded on a Varian Mercury Vx 400 spectrometer at 400 MHz and 100 MHz, respectively. Chemical shifts, *δ*, are reported in ppm relative to the internal standard TMS for both ^1^H and ^13^C NMR. *J* values are given in Hz. Elemental analyses were measured using a Perkin-Elmer 2400 C Elemental Analyzer. The fluorescence spectra were performed using a Cary Eclipse fluorescence spectrophotometer. UV-vis spectra were recorded on a JASCO-V570 spectrometer. EI mass spectra were recorded on a VG ZAB-HS mass spectrometer (VG, U.K.). IR spectra (KBr) were taken on a Bruker Equinox 55 spectrometer.

### Synthesis of 1,8-bis(2′-chloroacetyl)diaminonaphthalene

A suspension of 1,8-diaminonaphthalene (10.000 g, 63.2 mmol) and triethylamine (21.0 mL, 151.6 mmol) in CH_2_Cl_2_ (120 mL) was stirred for 30 min at 0 °C. Then chloroacetyl chloride (11.4 mL, 151.7 mmol) was dropwise added to the suspension above and stirred continually for 3 h at ambient temperature. The mixture was filtered and washed by water to afford 1,8-bis(2′-chloroacetyl)diaminonaphthalene as a yellow powder. Yield: 15.731 g (80%). M.p.: 265–267 °C. ^1^H NMR (400 MHz, DMSO-*d*_6_): *δ* 4.36 (s, 4H, C*H*_2_), 7.52 (t, *J* = 3.4 Hz, 6H, Ph*H*), 7.90 (t, *J* = 4.6Hz, 2H, Ph*H*), 10.10 (s, 2H, N*H*). ^13^C NMR (100 MHz, DMSO-*d*_6_): *δ* 43.8 (*C*H_2_), 126.0 (Ph*C*), 127.8 (Ph*C*), 132.18 (Ph*C*), 135.9 (Ph*C*), 165.6 (*C*=O).

### Preparation of 1,8-bis[2′-(*N*-ethylimidazoliumyl)acetylamino]naphthalene chloride (L^1^H_4_·Cl_2_)

A solution of *N*-ethyl-imidazole (1.538 g, 16.0 mmol) and 1,8-bis(2′-chloroacetylamino)naphthalene (2.000 g, 6.4 mmol) in DMF (150 mL) was heated to reflux for 7 days with stirring, and precipitated a black powder. The precipitate was collected by filtration and washed with a small portion of DMF to give 1,8-bis[2′-(*N*-ethyl-imidazoliumyl)acetylamino]naphthalene chloride. Yield: 1.480 g (48%). M.p.: 260–261 °C. Anal. Calcd for C_24_H_28_N_6_O_2_Cl_2_: C, 57.25; H, 5.60; N, 16.69%. Found: C, 57.20; H, 5.56; N, 16.68%. ^1^H NMR (400 MHz, DMSO-*d*_6_): *δ* 1.48 (t, *J* = 7.2 Hz, 6H, C*H*_3_), 4.32 (m, 4H, C*H*_2_), 5.50 (s, 4H, C*H*_2_), 7.59 (s, 2H, Ph*H*), 7.92 (t, *J* = 15.6 Hz, 4H, Ph*H*), 9.47 (s, 2H, 2-imi*H*), 11.07 (s, 2H, N*H*). ^13^C NMR (100 MHz, DMSO-*d*_6_): *δ* 15.6 (*C*H_3_), 44.7 (*C*H_2_), 52.1 (*C*H_2_), 121.6 (Ph*C*), 124.7 (Ph*C*), 125.8 (Ph*C*), 126.8 (Ph*C*), 127.9 (Ph*C*), 131.4 (Ph*C*), 136.0 (Ph*C*), 137.7 (Ph*C*), 164.9 (*C*=O) (imi = imidazolium).

### Preparation of 1,8-bis[2′-(*N*-^n^butyl-imidazoliumyl)acetylamino]naphthalene chloride (L^2^H_4_·Cl_2_). L^2^H_4_·Cl_2_

Was prepared according to the methods of **L**^**1**^**H**_**2**_**·Cl**_**2**_, only *N*-ethyl-imidazole was replaced by *N*-^n^butyl-imidazole (1.984 g, 16.0 mmol). Yield: 1.790 g (50%). M.p.: 240–242 °C. Anal. Calcd for C_28_H_36_N_6_O_2_Cl_2_: C, 60.10; H, 6.48; N, 15.01%. Found: C, 60.22; H, 6.32; N, 15.23%. ^1^H NMR (400 MHz, DMSO-*d*_6_): *δ* 0.93 (s, 6H, C*H*_3_), 1.30 (m, 4H, C*H*_2_), 1.82 (s, 4H, C*H*_2_), 4.28 (s, 4H, C*H*_2_), 5.55 (s, 4H, C*H*_2_), 7.59 (t, *J* = 7.4 Hz, 4H, Ph*H*), 7.97 (m, 6H, Ph*H*), 9.50 (s, 2H, 2-imi*H*), 11.16 (s, 2 H, N*H*). ^13^C NMR (100 MHz, DMSO-*d*_6_): *δ* 13.7 (*C*H_3_), 19.2 (*C*H_2_), 31.8 (*C*H_2_), 49.0 (*C*H_2_), 52.2 (*C*H_2_), 121.9 (Ph*C*), 124.8 (Ph*C*), 125.8 (Ph*C*), 126.8 (Ph*C*), 127.8 (Ph*C*), 131.4 (Ph*C*), 136.0 (Ph*C*), 138.0 (Ph*C*), 164.9 (*C*=O).

### Preparation of [L^1^H_2_Ag]Cl (1)

The mixture of **L**^**1**^**H**_**4**_**·Cl**_**2**_ (0.100 g, 0.2 mmol) and Ag_2_O (0.046 g, 0.2 mmol) in DMSO (2.5 mL) and CH_3_CN (12.5 mL) was heated to reflux for 24 h with stirring. After filtration, the solvent was evaporated to 5 mL, and the yellow powder of **1** was obtained after adding 5 mL of diethyl ether. Yield: 0.040 g (36%). M.p.: 192–194 °C. Anal. Calcd for C_24_H_26_AgN_6_O_2_Cl: C, 50.23; H, 4.56; N, 14.64%. Found: C, 50.44; H, 4.42; N, 14.52%. ^1^H NMR (400 MHz, DMSO-*d*_6_): *δ* 1.43 (t, *J* = 17.5 Hz, 6H, C*H*_3_), 4.20 (q, 4H, C*H*_2_), 5.06 (s, 4H, C*H*_2_), 7.29 (m, 4H, Ph*H*), 7.60 (d, *J* = 88 Hz, 4H, Ph*H*), 8.34 (s, 2 H, Ph*H*), 9.29 (s, 2H, N*H*). ^13^C NMR (100 MHz, DMSO-*d*_6_): *δ* 17.3 (*C*H_3_), 46.2 (*C*H_2_), 121.0 (*C*H_2_), 124.1 (Ph*C*), 125.5 (Ph*C*), 135.9 (Ph*C*), 166.3 (*C*=O).

### Preparation of [L^1^Ni] (2)

NiCl_2_ (0.052 g, 0.4 mmol) was mixed with **L**^**1**^**H**_**4**_**·Cl**_**2**_ (0.100 g, 0.2 mmol) and K_2_CO_3_ (0.138 g, 1.0 mmol) in DMSO (2.5 mL) and CH_3_CN (12.5 mL), and the reaction kept going for 24 h at 60 °C with stirring. After filtration, the solvent was evaporated to 5 mL, and the pale yellow powder of **2** was obtained after adding 5 mL of diethyl ether. Yield: 0.040 g (40%). M.p.:>320 °C. Anal. Calcd for C_24_H_24_NiN_6_O_2_: C, 59.16; H, 4.96; N, 17.25%. Found: C, 59.32; H, 4.87; N, 17.43%. ^1^H NMR (400 MHz, DMSO-*d*_6_): *δ* 1.06 (t, *J* = 7.2 Hz, 6H, C*H*_3_), 3.41 (q, *J* = 6.9 Hz, 4H, C*H*_2_), 4.50 (t, *J* = 3.2 Hz, 4H, C*H*_2_), 6.70 (s, 2H, Ph*H*), 7.11 (t, *J* = 7.6 Hz, 2H, Ph*H*), 7.28 (d, *J* = 2.0 Hz, 2H, Ph*H*), 7.40 (d, *J* = 3.0 Hz, 2H, Ph*H*), 7.55 (d, *J* = 0.5 Hz, 2H, Ph*H*). ^13^C NMR (100 MHz, DMSO-*d*_6_): *δ* 15.6 (*C*H_3_), 44.5 (*C*H_2_), 65.3 (*C*H_2_), 121.5 (Ph*C*), 122.2 (Ph*C*), 124.5 (Ph*C*), 135.4 (Ph*C*), 166.6 (*C*=O), 175.0 (2-imi*C*).

### Preparation of [L^2^Ni] (3)

[L^2^Ni] (**3**) was prepared according to the methods of **2**, only **L**^**1**^**H**_**4**_**·Cl**_**2**_ was replaced by **L**^**2**^**H**_**4**_**·Cl**_**2**_ (0.100 g, 0.2 mmol). Yield: 0.020 g (20%). M.p.: >320 °C. Anal. Calcd for C_28_H_32_NiN_6_O_2_: C, 61.90; H, 5.93; N, 15.46%. Found: C, 61.78; H, 5.84; N, 15.58%. ^1^H NMR (400 MHz, DMSO-*d*_6_): *δ* 0.70 (t, *J* = 23 Hz, 6H, C*H*_3_), 1.07 (m, 4H, C*H*_2_), 1.44 (m, 4H, C*H*_2_), 3.80 (t, *J* = 48.4 Hz, 4H, C*H*_2_), 5.03 (s, 4H, C*H*_2_), 6.78 (s, 2H, Ph*H*), 7.09 (t, *J* = 7.8 Hz, 2H, Ph*H*), 7.29 (d, *J* = 8.0 Hz, 2H, Ph*H*), 7.36 (s, 2H, Ph*H*), 7.55 (d, *J* = 0.8 Hz, 2H, Ph*H*). ^13^C NMR (100 MHz, DMSO-*d*_6_): *δ* 13.3 (*C*H_3_), 19.1 (*C*H_2_), 30.3 (*C*H_2_), 49.5 (*C*H_2_), 53.7 (*C*H_2_), 112.4 (Ph*C*), 112.5 (Ph*C*), 113.2 (Ph*C*), 116.0 (Ph*C*), 121.7 (Ph*C*), 122.2 (Ph*C*), 123.9 (Ph*C*), 132.0 (Ph*C*), 165.1 (*C*=O), 175.0 (2-imi*C*).

### Preparation of [L^1^H_2_Hg(HgCl_4_)] (4)

HgCl_2_ (0.110 g, 0.4 mmol) was mixed with **L**^**1**^**H**_**4**_**·Cl**_**2**_ (0.100 g, 0.2 mmol) and KOBu^t^ (0.056 g, 0.5 mmol) in DMSO (2.5 mL) and CH_3_CN (12.5 mL). The solution was heated to 80 °C for 24 h with stirring. After filtration, the solvent was evaporated 10 mL, and the pale brown powder of **4** was obtained after adding 5 mL of diethyl ether. Yield: 0.080 g (40%). M.p.: > 320 °C. Anal. Calcd for C_24_H_26_Hg_2_N_6_O_2_Cl_4_: C, 29.61; H, 2.69; N, 8.63%. Found: C, 29.76; H, 2.58; N, 8.77%. ^1^H NMR (400 MHz, DMSO-*d*_6_): δ 1.46 (t, *J* = 7.2 Hz, 6H, C*H*_3_), 4.56 (m, 4H, C*H*_2_), 5.57 (s, 4H, C*H*_2_), 7.51 (d, *J* = 4.8 Hz, 4H, Ph*H*), 7.77 (d, *J* = 18.8 Hz, 4H, Ph*H*), 7.88 (t, *J* = 6.4 Hz, 4H, Ph*H*), 10.22 (s, 2H, N*H*). ^13^C NMR (100 MHz, DMSO-*d*_6_): *δ* 16.0 (*C*H_3_), 45.6 (*C*H_2_), 52.7 (*C*H_2_), 122.2 (Ph*C*), 125.0 (Ph*C*), 125.4 (Ph*C*), 125.5 (Ph*C*), 127.1 (Ph*C*), 131.8 (Ph*C*), 135.4 (Ph*C*), 165.0 (*C*=O), 176.8 (2-imi*C*).

### Fluorescence titrations

The stock solution (1.0 × 10^−4^ M) of the host was prepared and diluted to the suitable concentration with CH_3_CN. The stock solutions (1.0 × 10^−3^ M or 1.0 × 10^−4^ M) of guest were prepared and diluted in the same solvent. Test solutions were prepared through placing 0.2 mL of host stock solution into a 10 mL volumetric flask, and the appropriate amount of the stock solutions (1.0 × 10^−3^ M or 1.0 × 10^−4^ M) of guest were added with a microsyringe. The mixture solutions were diluted to 10 mL with CH_3_CN to prepare test solutions. The concentrations of guest in the test solutions were from 0 to 24.0 × 10^−6^ M, and the concentration of host stayed the same (2.0 × 10^−6^ M). The test solutions were kept at 25 °C for 8–10 minutes, and then fluorescence spectra were recorded with the excitation wavelength at 330 nm, and the excitation and emission slits are 5 nm and 5 nm. Statistical analysis of the data was carried out using Origin 8.0. CH_3_CN used in the titrations was freshly distilled.

### Quantum yields

Fluorescence quantum yields (Φ) of **L**^**1**^**H**_**4**_**·Cl**_**2**_ and complex **1** were determined by using 1-aminonaphthalene (Φ = 0.39) in CH_3_CN as the standard compound. Fluorescence quantum yields could be calculated according to the equation (2) below^64^.

2 $${{\rm{\Phi }}}_{{\rm{U}}}={{\rm{\Phi }}}_{{\rm{S}}}({A}_{S}/{A}_{U})({F}_{U}/{F}_{S}){({{\rm{n}}}_{{\rm{U}}}/{{\rm{n}}}_{{\rm{S}}})}^{2}$$

where Φ_U_, *A*_U_ and *F*_U_ are the quantum yield, the absorbance and the emission intensity for **L**^**1**^**H**_**4**_**·Cl**_**2**_ or complex **1**. Φ_S_, *A*_S_ and *F*_S_ are the quantum yield, the absorbance and the emission intensity for 1-aminonaphthalene. n_U_ and n_S_ are the average refractive index of the sample solution (n_U_ = n_S_ = n_acetonitrile_).

### Method for Job’s plot

The stock solution (1.0 × 10^−4^ M) of the host was prepared and diluted to the suitable concentration with CH_3_CN. The stock solutions (1.0 × 10^−4^ M or 1.0 × 10^−3^ M) of guest were prepared and diluted in the same solvent. The molar fractions of host and guest in the test solutions were from 1 to 0 and 0 to 1, respectively. The total concentration is 4.0 × 10^−5^ M and different amounts of host and guest solutions were placed into a 10 mL volumetric flask using a microsyringe, and then diluted to 10 mL. The test solutions were kept at 25 °C for 8–10 minutes, and then absoption spectra were measured. Statistical analysis of the data was carried out using Origin 8.0.

### X-Ray data collection and structure determinations

A Bruker Apex II CCD diffractometer were used for the collection of diffraction data of **1**–**4**^78^. The structure was solved with the SHELXS program^79^. Figures 1–4 were formed via employing Crystal-Maker^80^. Other details for structural analysis and crystallographic data was listed in Tables 1 and 2.

**Table 1:** Summary of crystallographic data for **1–4**.

	**1**	**2**·Et_2_O
Chemical formula	C_24_H_26_AgClN_6_O_2_	C_24_H_24_N_6_NiO_2_·Et_2_O
Formula weight	573.83	561.32
Cryst syst	Monoclinic	Monoclinic
Space group	*P*2_1_	*P*2_1_/*c*
*a*, Å	4.555(3)	11.321(1)
*b*, Å	20.043(1)	12.999(2)
*c*, Å	14.775(1)	18.718(3)
*α*, deg	90	90
*β*, deg	91.2(1)	104.3(3)
*γ*, deg	90	90
*V*, Å^3^	1348.8(1)	2668.1(7)
*Z*	2	4
*D*_calcd_, Mg m^−3^	1.413	1.397
Abs coeff, mm^−1^	0.877	0.769
*F*(000)	584	1184
Cryst size, mm	0.14 × 0.12 × 0.11	0.18 × 0.17 × 0.16
*θ*_min_, *θ*_max_, deg	2.03, 25.01	1.86, 25.01
*T*, K	173(2)	173(2)
No. of data collected	7781	13811
No. of unique data	3736	4669
No. of refined params	335	360
Goodness-of-fit on *F*^2a^	1.092	1.071
**Final** ***R*** **indices**^**b**^ **[*I*** > **2*σ*(*I*)]**		
*R*_1_	0.0462	0.0775
*wR*_2_	0.1236	0.2053
***R*** **indices (all data)**		
*R*_1_	0.0511	0.0965
*wR*_2_	0.1279	0.2244

^a^*GOF* = [Σ*w(F*_o_^2^ *− F*_c_^2^*)*^2^/(*n* − *p*)]^1/2^, where *n* is the number of reflection and *p* is the number of parameters refined. ^b^*R*_1_ = Σ(*||F*_o_*| − |F*_c_*||*)/Σ*|F*_o_*|*; *wR*_2_ = [Σ[*w*(*F*_o_^2^ − *F*_c_^2^)^2^]/Σ*w*(*F*_o_^2^)^2^]^1/2^.

**Table 2:** Summary of crystallographic data for **3** and **4**.

	**3**·1.5CH_3_CN	**4**·CH_3_CN·DMSO
Chemical formula	C_28_H_32_N_6_NiO_2_	C_24_H_26_Cl_4_Hg_2_N_6_O_2_·CH_3_CN·DMSO
Formula weight	543.28	1092.67
Cryst syst	Orthorhombic	Monoclinic
Space group	*Pbca*	*P*2_1_/*n*
*a*, Å	17.192(5)	9.893(3)
*b*, Å	17.019(6)	24.332(6)
*c*, Å	17.565(6)	14.889(3)
*α*, deg	90	90
*β*, deg	90	97.4(2)
*γ*, deg	90	90
*V*, Å^3^	5139.7(4)	3554.1(1)
*Z*	8	4
*D*_calcd_, Mg m^−3^	1.404	2.042
Abs coeff, mm^−1^	1.392	18.929
*F*(000)	2288	2080
Cryst size, mm	0.25 × 0.24 × 0.20	0.25 × 0.15 × 0.14
*θ*_min_, *θ*_max_, deg	4.43, 67.07	3.50, 67.07
*T*, K	173(2)	173(2)
No. of data collected	13621	13064
No. of unique data	4591	6340
No. of refined params	336	409
Goodness-of-fit on *F*^2a^	1.020	1.080
**Final** ***R*** **indices** ^**b**^ **[** ***I*** ** > 2** ***σ*** **(** ***I*** **)]**		
*R*_1_	0.0393	0.0400
*wR*_2_	0.0785	0.1047
***R*** **indices (all data)**		
*R*_1_	0.0662	0.0440
*wR*_2_	0.0860	0.1087

^a^*GOF* = [Σ*w(F*_o_^2^ *− F*_c_^2^)^2^/(*n* − *p*)]^1/2^, where *n* is the number of reflection and *p* is the number of parameters refined. ^b^*R*_1_ = Σ(*||F*_o_*| − |F*_c_*||*)/Σ*|F*_o_*|*; *wR*_2_ = [Σ[*w*(*F*_o_^2^ − *F*_c_^2^)^2^]/ Σ*w*(*F*_o_^2^)^2^]^1/2^.

## Electronic supplementary material

Supplementary Information
