# Supplementary material for: Preparation of Macrometallocycle and Selective Sensor for Copper Ion
Source: Sci Rep. 2018 Jul 19;8:10943. doi: 10.1038/s41598-018-29356-z (PMC6053464; doi:10.1038/s41598-018-29356-z)
Supplement: Supplementary file 1 — Supplementary Information [file 41598_2018_29356_MOESM1_ESM.doc]

**Preparation of Macrometallocycle and Selective Sensor for Copper Ion**

Yingjie Liu,^a^ Zhixiang Zhao^b^, Qingxiang Liu^b*^

^a^*Tianjin Key Laboratory of Process Measurement and Control,* *Institute of Robotics and Autonomous Systems,* *Tianjin University, Tianjin* *300072, China.*

^b^*Key Laboratory of Inorganic-Organic Hybrid Functional Materials Chemistry (Tianjin Normal University), Ministry of Education; Tianjin Key Laboratory of Structure and Performance for Functional Molecules; College of Chemistry, Tianjin Normal University, Tianjin 300387, China.*

** Corresponding author, E-mail: tjnulqx@163.com*

**List of the contents**

1. CCDC numbers for complexes **1**-**4**

2. The dihedral angles of complexes **1**-**4** (Table S1)

3. The fluorescence and UV/Vis studies of complex **1** (Figures S1 and Figure S6)

4. The figures of high-resolution mass spectra (HRMS) of **1**·Cu^2+^ (Figure S7)

5. The infrared spectra of **1** and **1**·Cu^2+^ (Figure S8)

6. The ^1^H NMR and ^13^C NMR spectra of intermediates, precursors **L^1^H_4_·Cl_2_**-**L^2^H_4_·Cl_2_** and complexes **1-4** (Figure S9-Figure S22)

**1. CCDC numbers for complexes 1-4**

CCDC 1561891-1561894 contains the supplementary crystallographic data for complexes **1**-**4**. These data can be obtained free of charge via http//www.ccdc.cam.ac.uk/conts/retrieving.html, or from the Cambridge Crystallographic Data Centre, 12 Union Road, Cambridge, CB2 1EZ, UK; fax: (+44) 1223-336-033; or e-mail: deposit@ccdc.cam.ac.uk.

**2. The dihedral angles of complexes 1-4**

**Table S1.** In the same ligand of **1-4**, the dihedral angles (˚) between two imidazole rings (A) in the same NHC-metal-NHC units, and the dihedral angles (˚) between [naphthalene](app:ds:naphthalene) ring and two imidazole rings (B)

| Complexes | A | B |
| --- | --- | --- |
| **1** | 9.6(5) | 54.2(6), 60.7(6) |
| **2** | 83.4(3) | 56.1(5), 51.5(5) |
| **3** | 74.9(1) | 68.0(1), 75.9(8) |
| **4** | 14.2(4) | 55.7(2), 66.8(3) |

**3. The fluorescence and UV/Vis studies of complex 1**

**
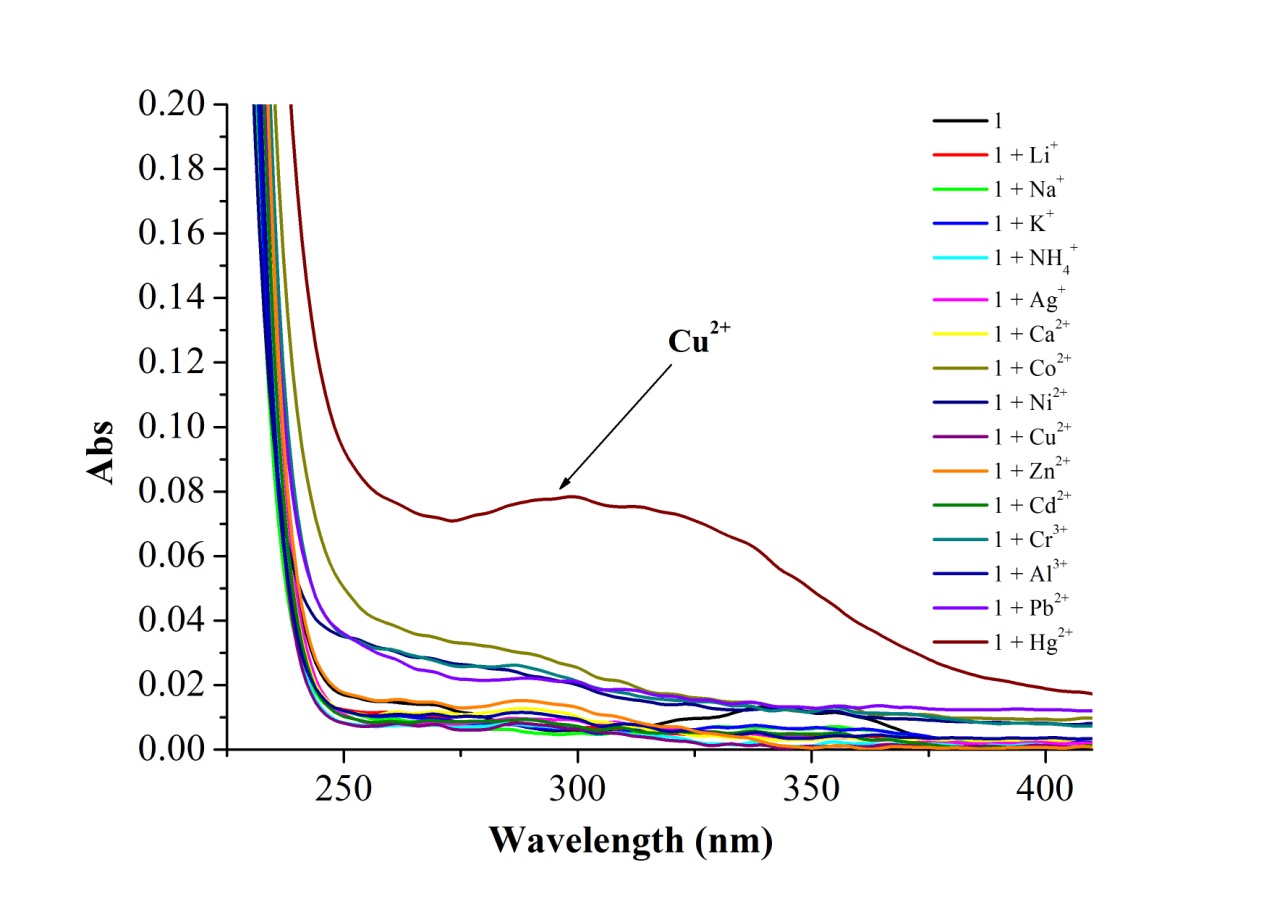
**

**Figure S1.** UV-vis absorption spectra of **1** (4.0 × 10^-6^ mol/L) and upon the addition of salts (20 × 10^-6^ mol/L) of Li^+^, Na^+^, K^+^, NH_4_^+^, Ag^+^, Ca^2+^, Co^2+^, Ni^2+^, Cu^2+^, Zn^2+^, Cd^2+^, Cr^3+^, Al^3+^, Pb^2+^ and Hg^2+^ in CH_3_CN at 25 ˚C.

**
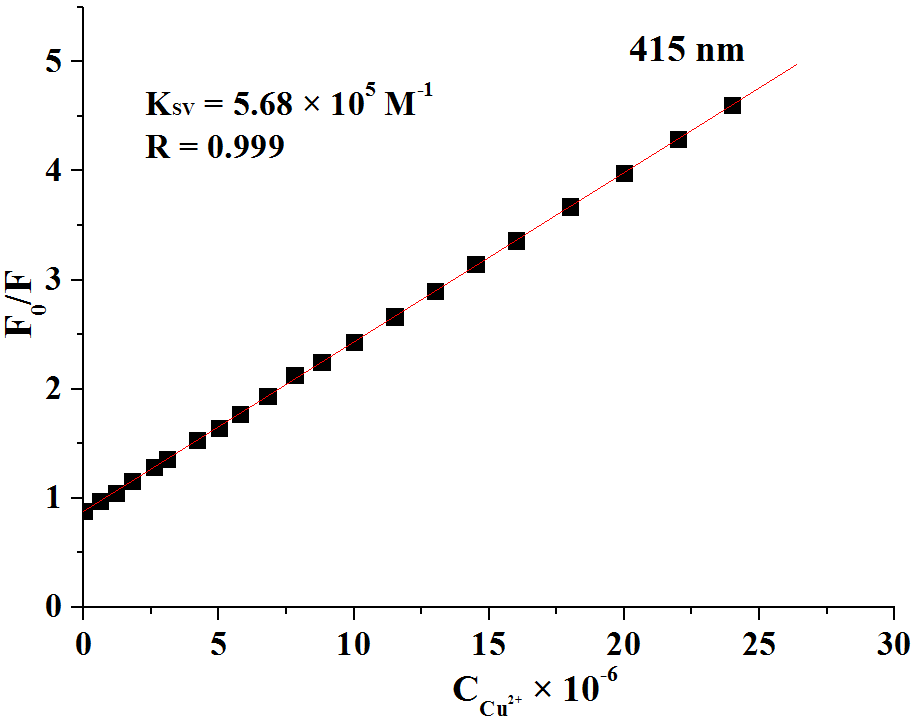
**

**Figure S2.** Stern-Volmer plot of host **1** quenched by Cu^2+^ in acetonitrile solutions at 415 nm. The *K_SV_* is 5.68 × 10^5^ M^-1^, and the linear range is from 0-24 × 10^-6^ mol/L.

**
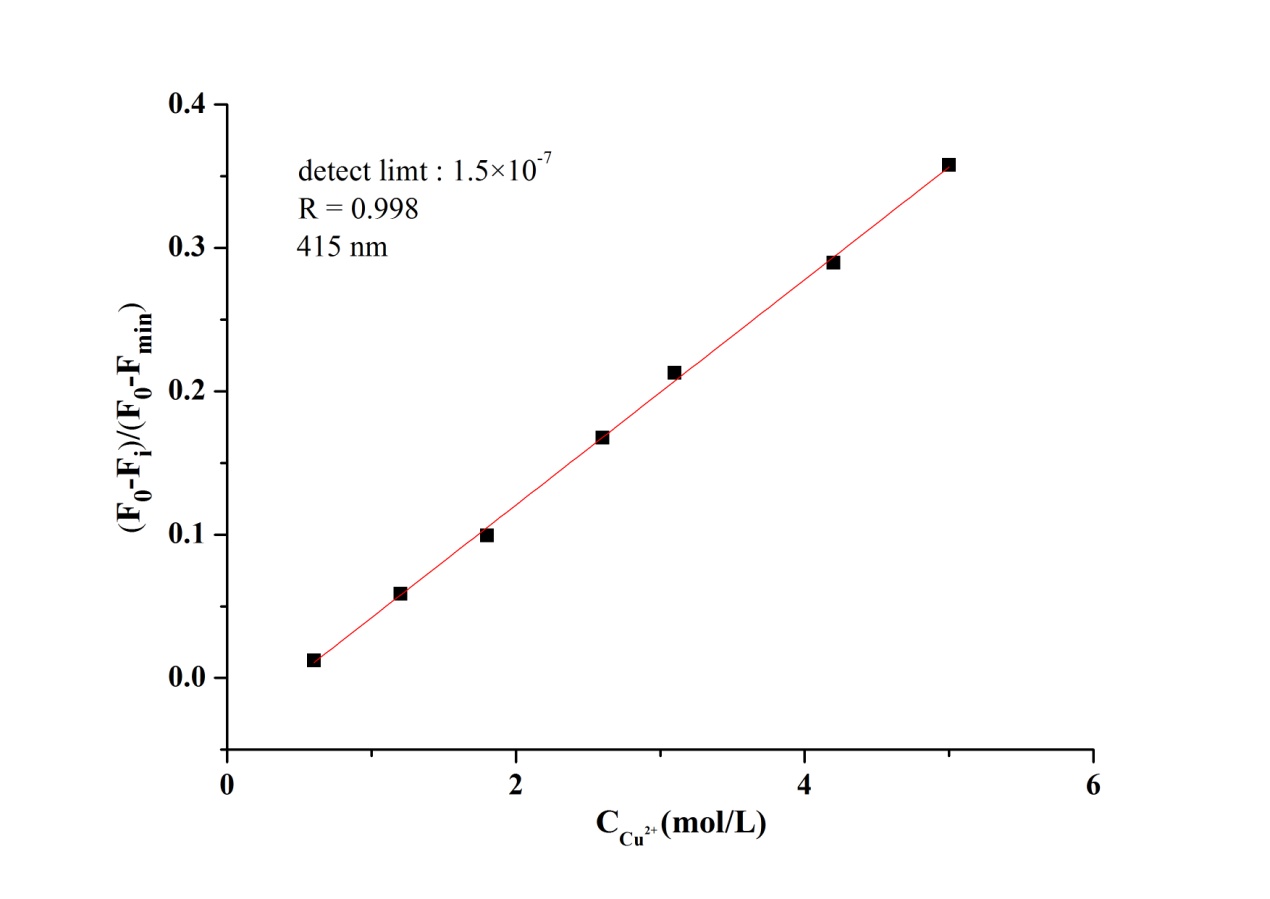
**

**Figure S3.** Emission (at 415 nm) of **1** at different concentrations of Cu^2+^ (0, 0.6, 1.2, 1.8, 2.6, 3.4, 4.2, 5.0, 5.8 μM) added, normalized between the minimum emission (0.0 μM Cu^2+^) and the emission (5.8 μM Cu^2+^). The detection limit was determined to be 2.0 × 10^-6^ mol/L.


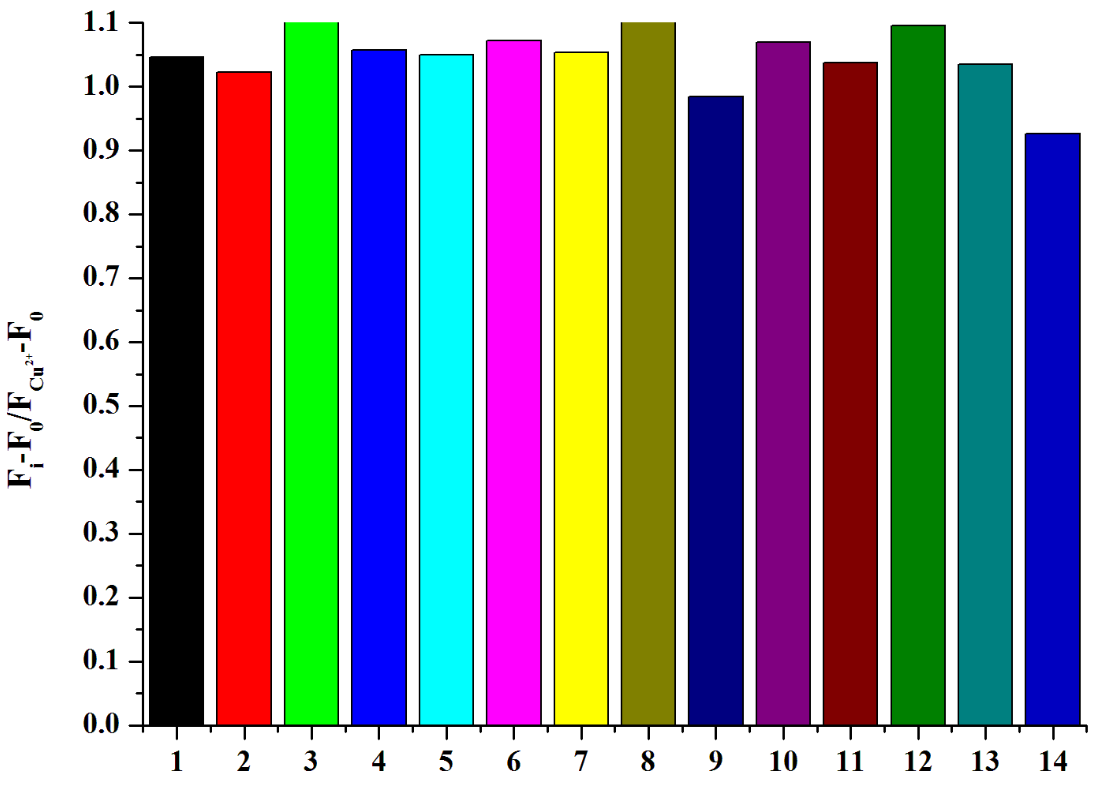


**Figure S4.** Change ratio (F_i_ - F_0_)/(F_Cu_^2+^ - F_0_) of fluorescence intensity of **1** (2.0 × 10^-6^ mol/L) at 415 nm in various mixtures of metal ions (Cu(NO_3_)_2_ 1.0 × 10^-5^ mol/L and another metal ion 1.0 × 10^-5^ mol/L). Background cations. 1: Cu^2+^ + Li^+^; 2: Cu^2+^ + Na^+^; 3: Cu^2+^ + K^+^; 4: Cu^2+^ + NH_4_^+^; 5: Cu^2+^ + Ag^+^; 6: Cu^2+^ + Ca^2+^; 7: Cu^2+^ + Co^2+^; 8: Cu^2+^ + Ni^2+^ ; 9: Cu^2+^ + Zn^2+^; 10: Cu^2+^ + Cd^2+^ ; 11: Cu^2+^ + Cr^3+^; 12: Cu^2+^ + Al^3+^; 13: Cu^2+^ + Pb^2+^; 14: Cu^2+^ + Hg^2+^; in CH_3_CN at 25 ˚C.


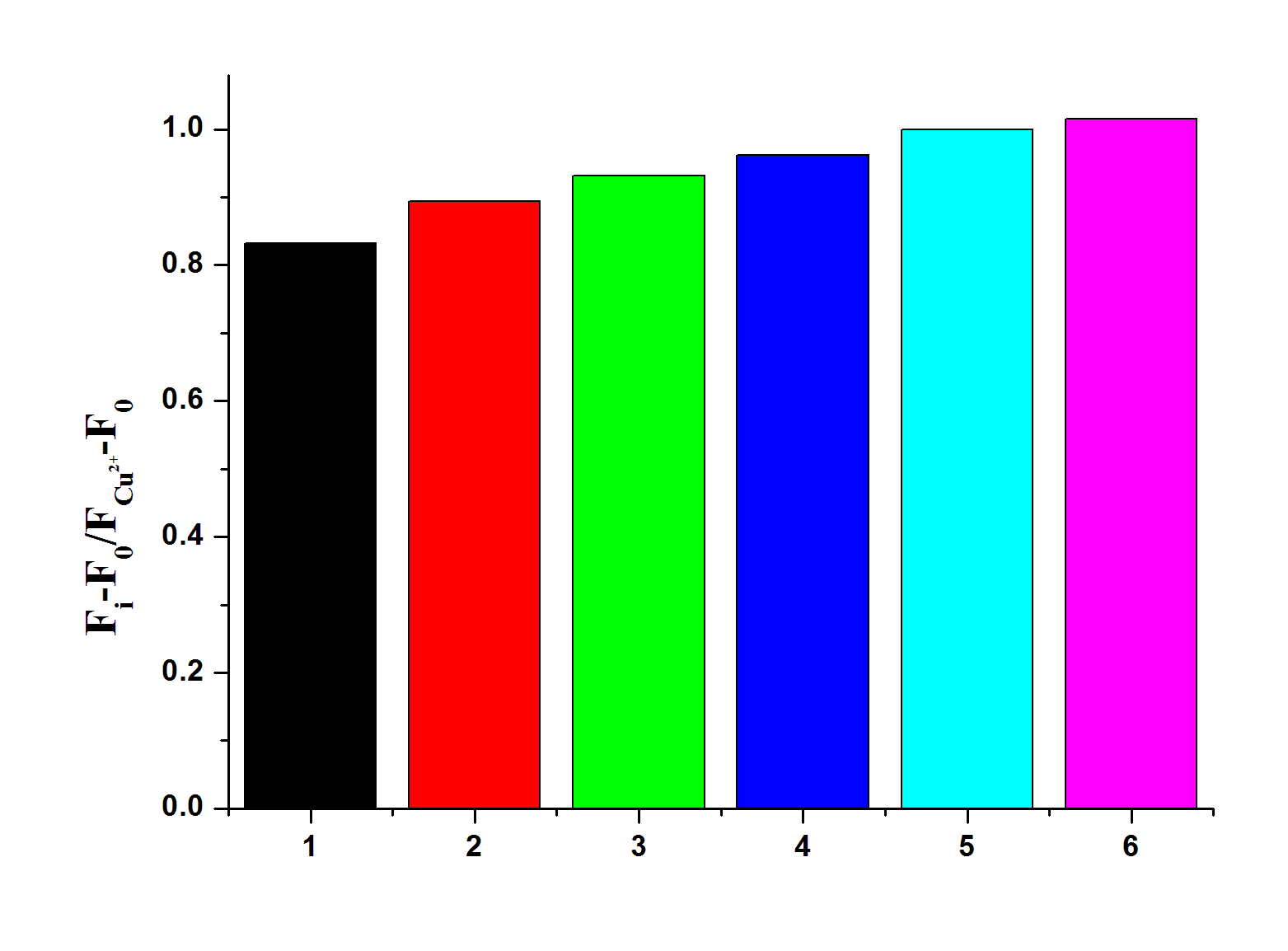


**Figure S5.** Change ratio (F_i_ - F_0_)/(F_Cu_^2+^ - F_0_) of fluorescence intensity of **1** (2.0 × 10^-6^ mol/L) at 415 nm upon addition of copper(II) salts (1.0 × 10^-5^ mol/L) with different counter anions (Br^-^, SO_4_^2-^, OAc^-^, Cl^-^, NO_3_^-^ and CO_3_^2-^).

**
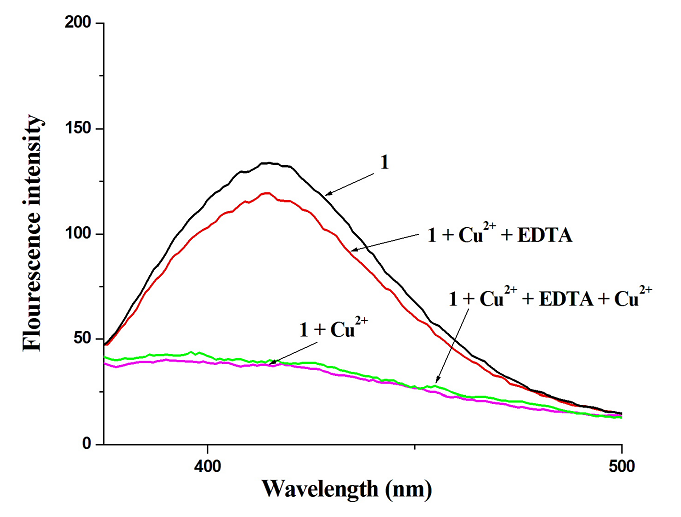
**

**Figure S6.** Fluorescence reversibility of **1** upon the detection of Cu^2+^. Fluorescent change of **1** after the addition of Cu^2+^ and the sequential addition of an EDTA and Cu^2+^ aqueous solution, respectively.

**4. The figures of high-resolution mass spectra (HRMS) of 1·Cu^2+^**


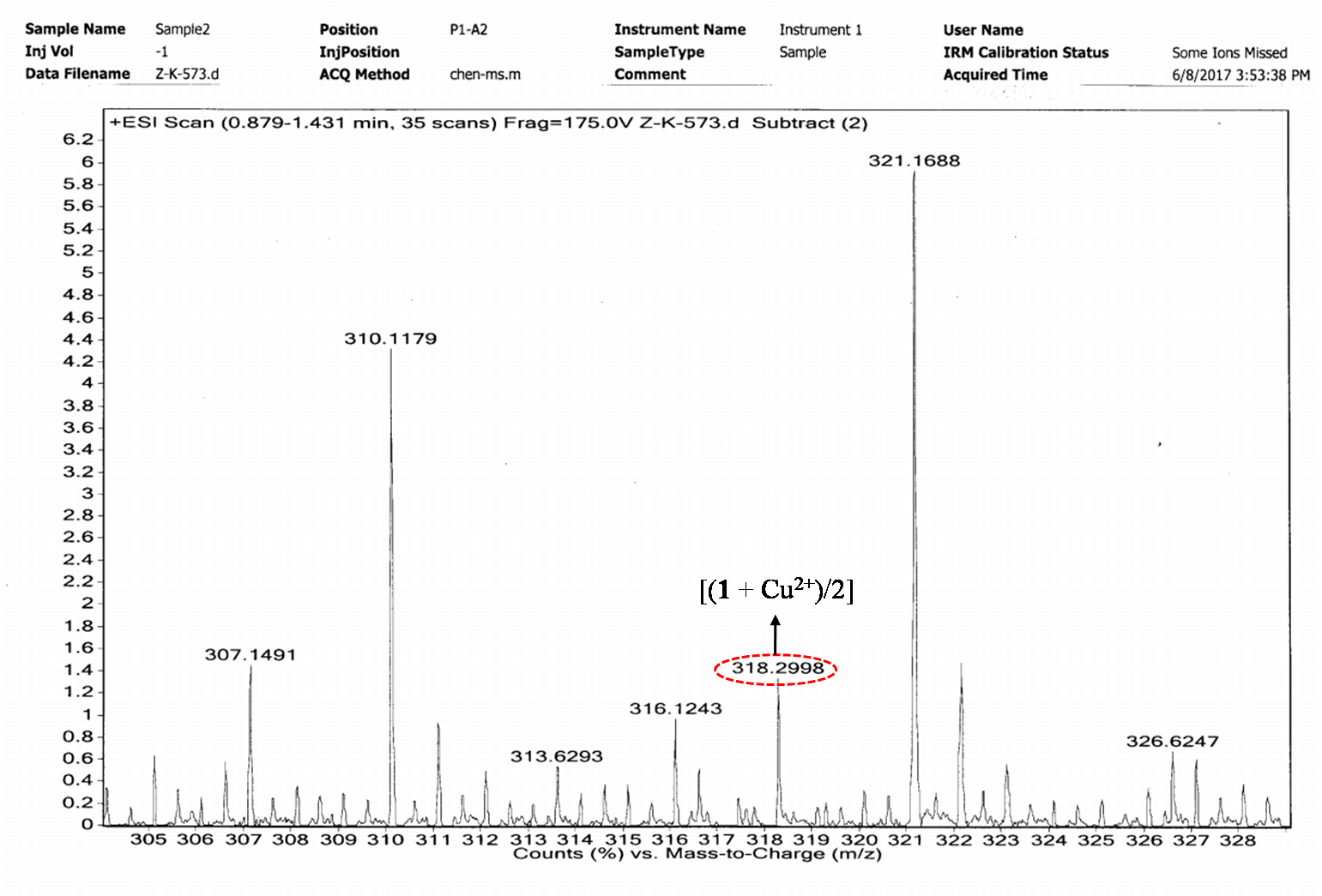


**Figure S7.** Mass spectrum of **1**·Cu^2+^. MS (EI): m/z {[(**1**·Cu^2+^)/2]}^+^ = 318.2998.

**5. The infrared spectra of 1 and 1·Cu^2+^**


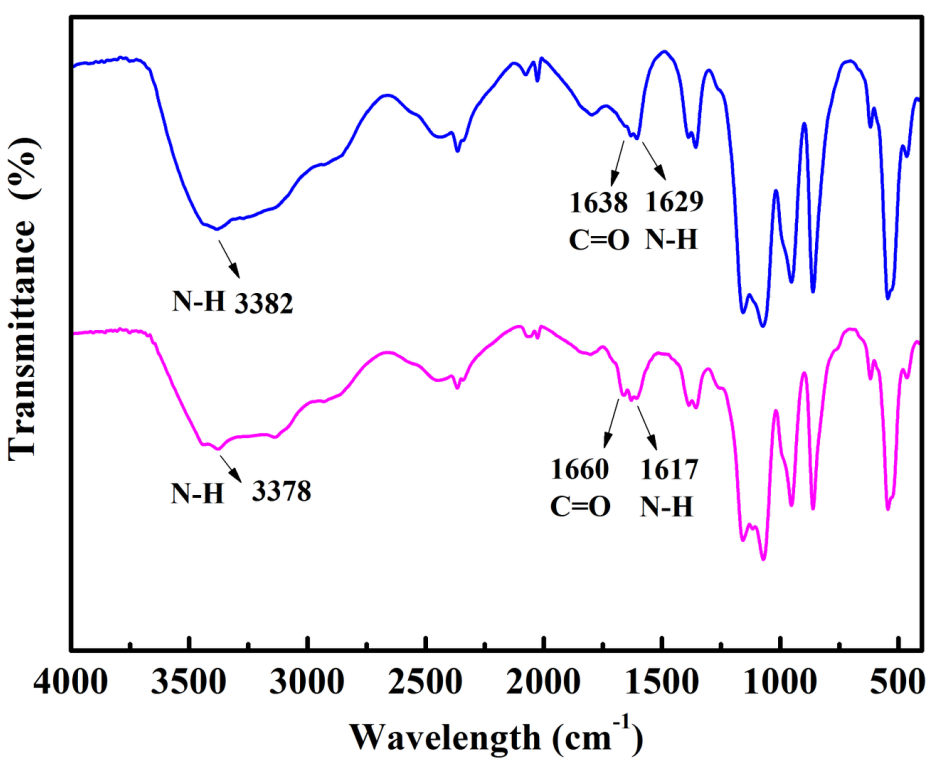


**Figure S8.** Infrared spectroscopy of **1·**Cu^2+^ (top) and free **1** (bottom).

**6. The ^1^H NMR and ^13^C NMR spectra of intermediates, precursors L^1^H_4_·Cl_2_-L^2^H_4_·Cl_2_ and complexes 1-4**


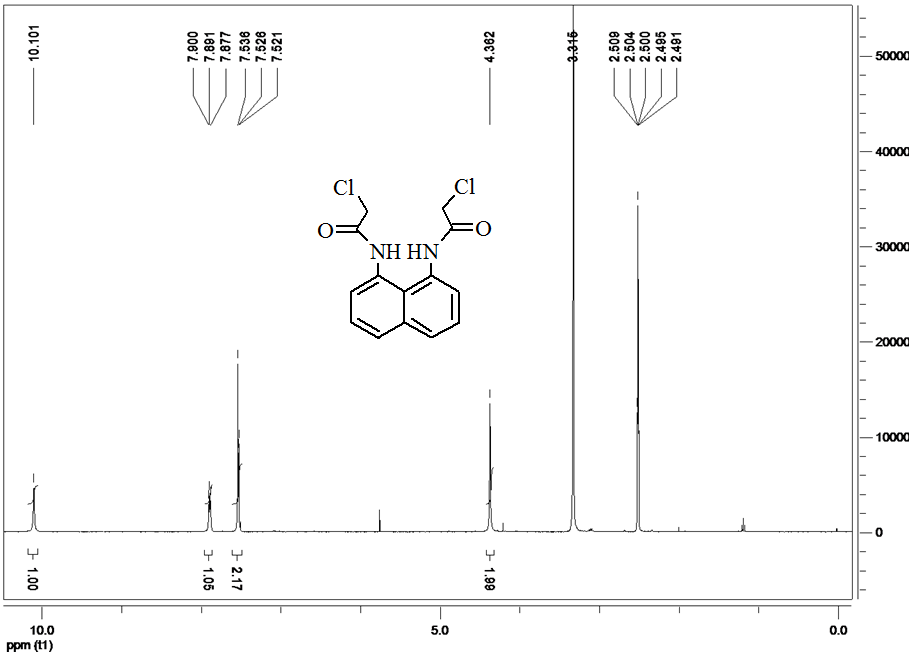


**Figure S9.** The ^1^H NMR (400 MHz, DMSO-*d*_6_) spectra of 1,8-bis(2’-chloroacetyl)diaminonaphthalene.


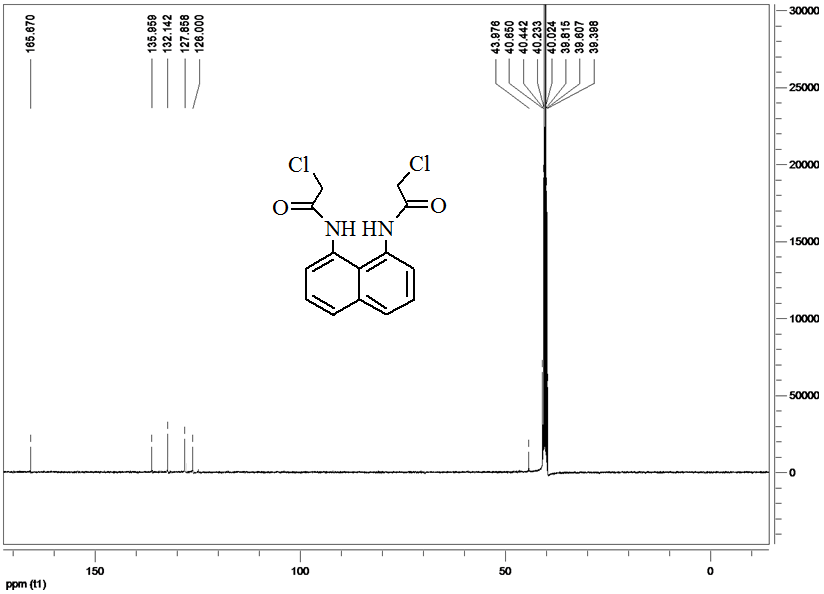


**Figure S10.** The ^13^C NMR (100 MHz, DMSO-*d*_6_) spectra of 1,8-bis(2’-chloroacetyl)diaminonaphthalene.


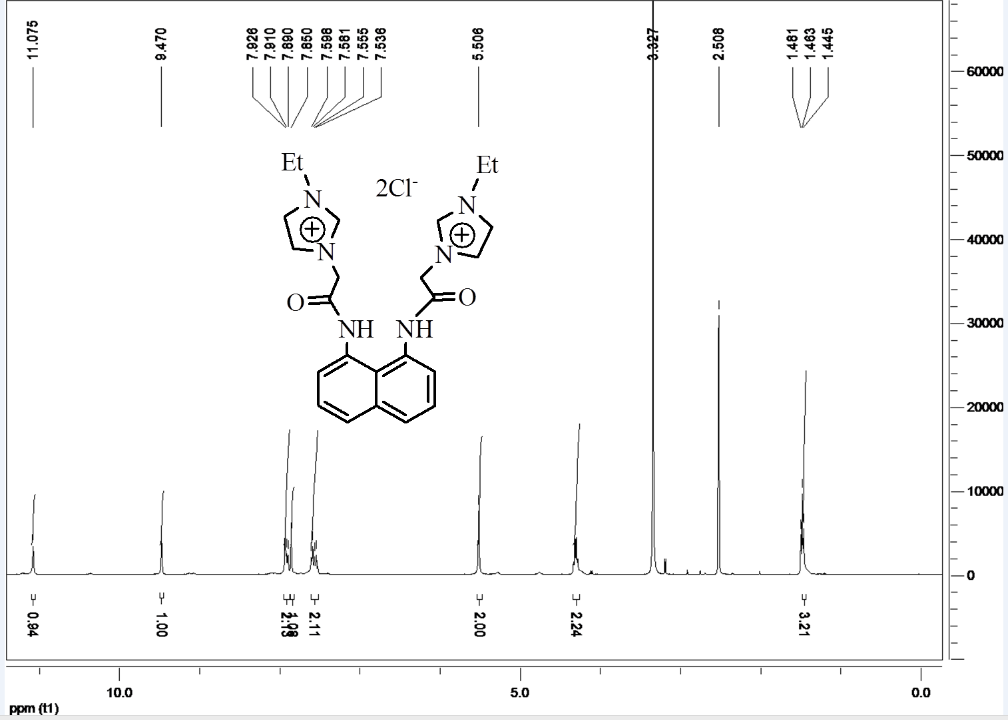


**Figure S11.** The ^1^H NMR (400 MHz, DMSO-*d*_6_) spectra of **L^1^H_4_·Cl_2._**


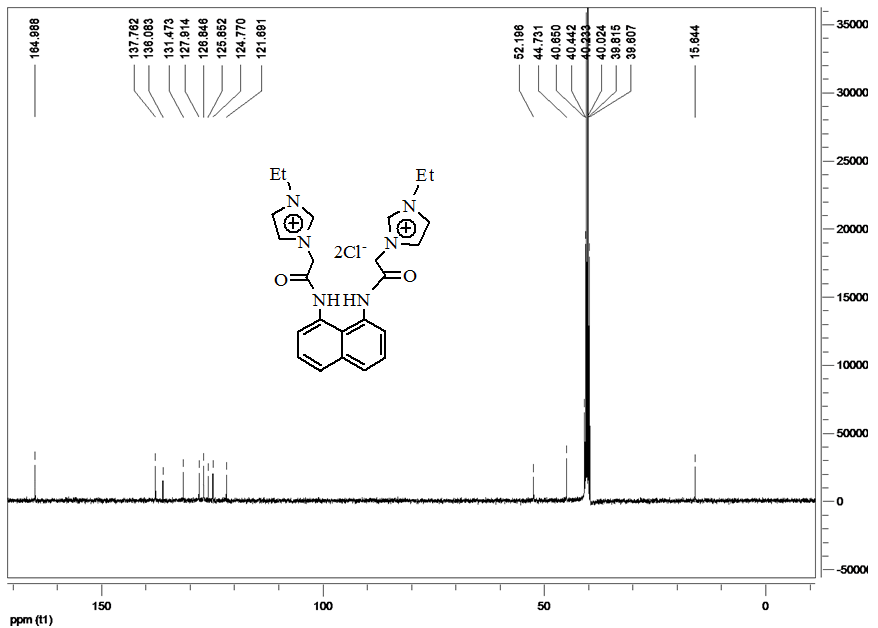


**Figure S12.** The ^13^C NMR (100 MHz, DMSO-*d*_6_) spectra of **L^1^H_4_·Cl_2._**


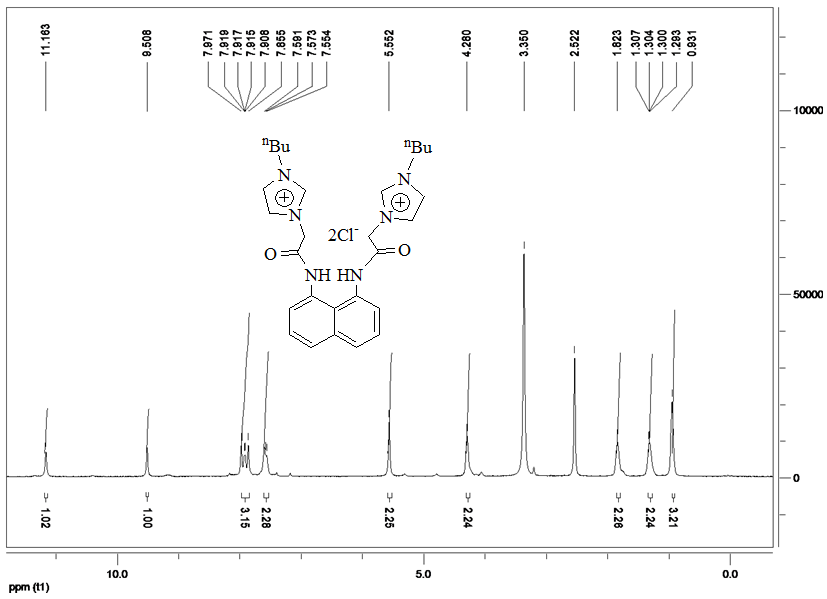


**Figure S13.** The ^1^H NMR (400 MHz, DMSO-*d*_6_) spectra of **L^2^H_4_·Cl_2._**


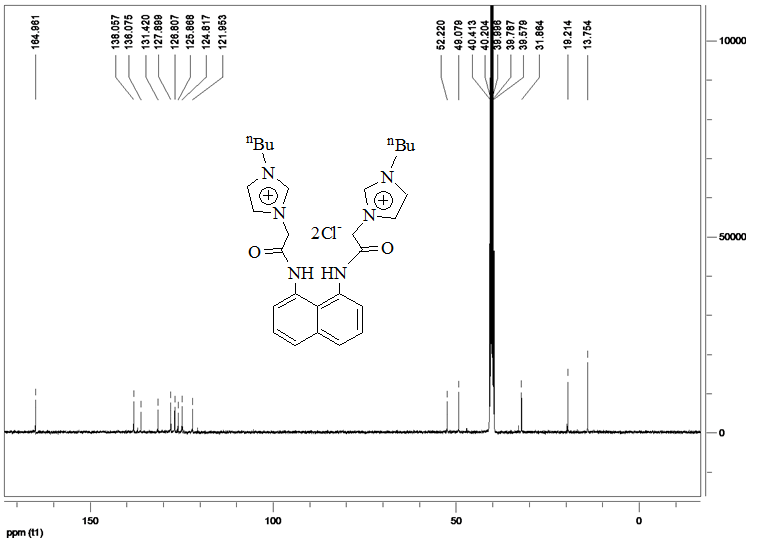


**Figure S14.** The ^13^C NMR (400 MHz, DMSO-*d*_6_) spectra of **L^2^H_4_·Cl_2._**


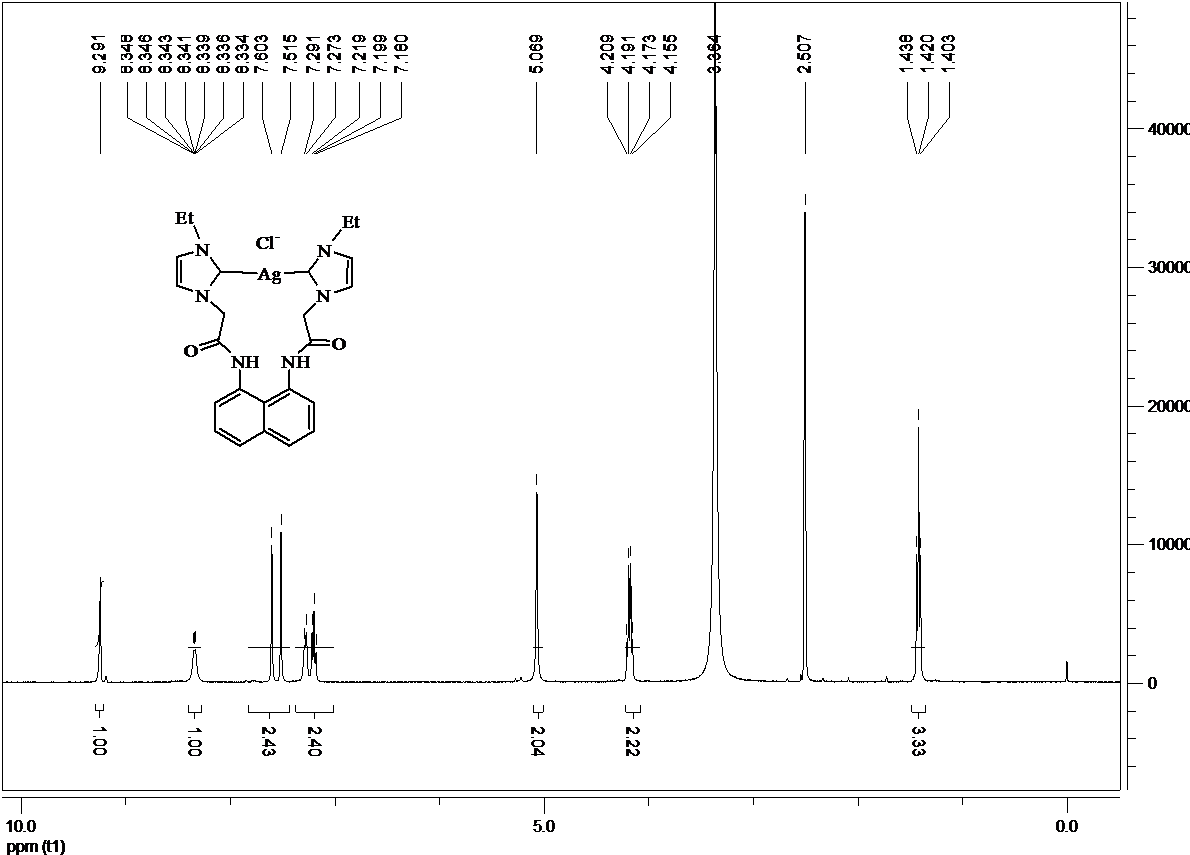


**Figure S15.** The ^1^H NMR (400 MHz, DMSO-*d*_6_) spectra of complex **1**.


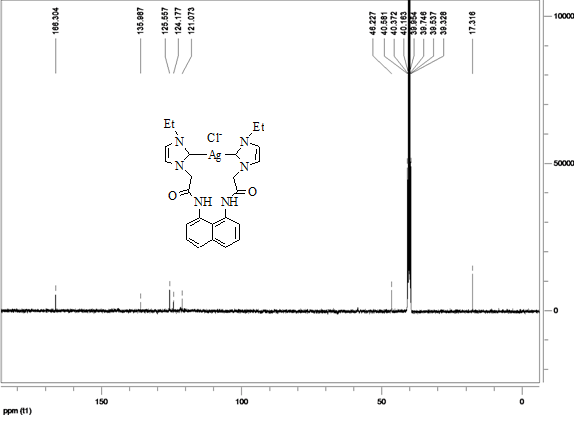


**Figure S16.** The ^13^C NMR (100 MHz, DMSO-*d*_6_) spectra of complex **1**.

**
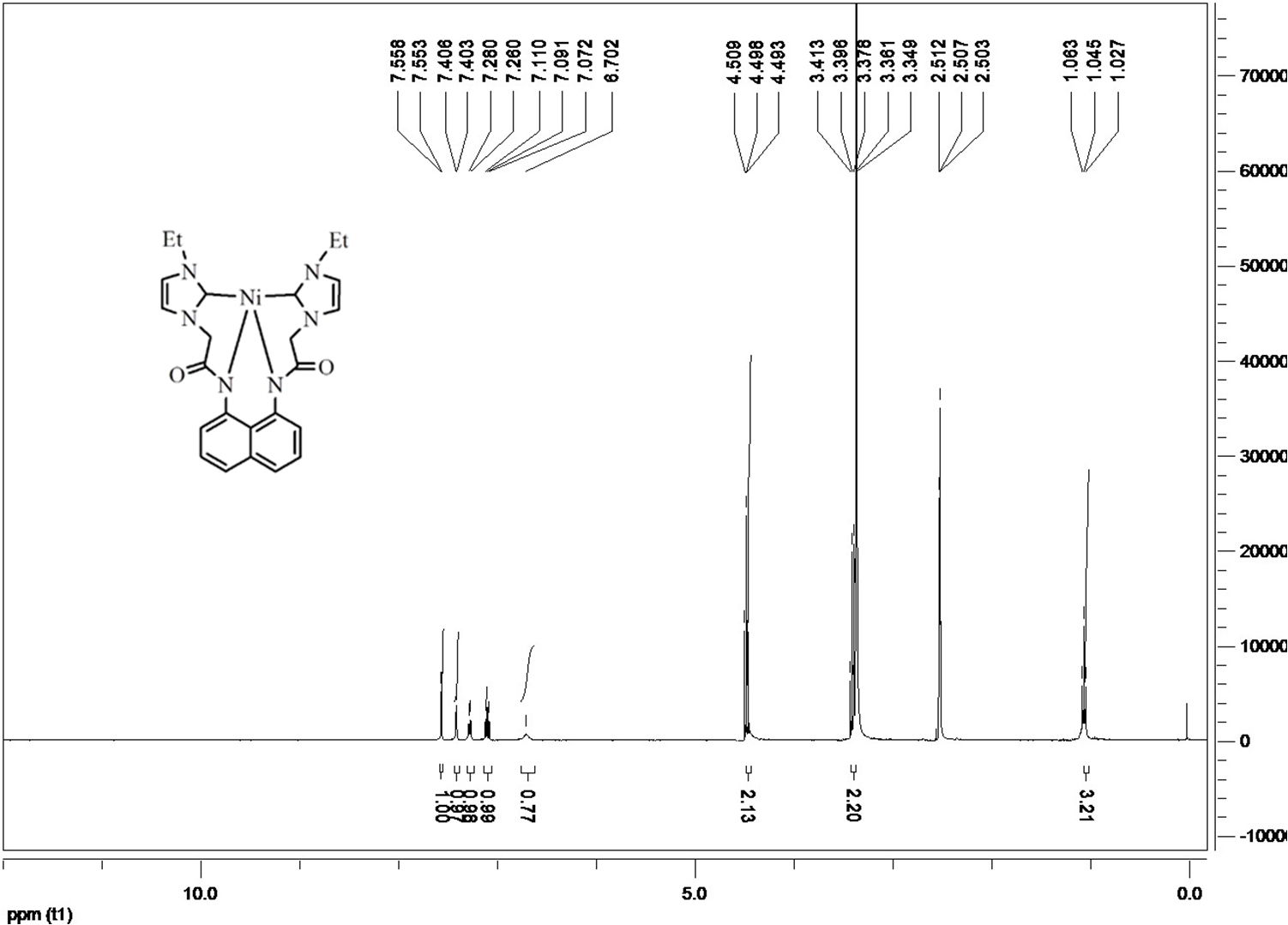
**

**Figure S17.** The ^1^H NMR (400 MHz, DMSO-*d*_6_) spectra of complex **2**.


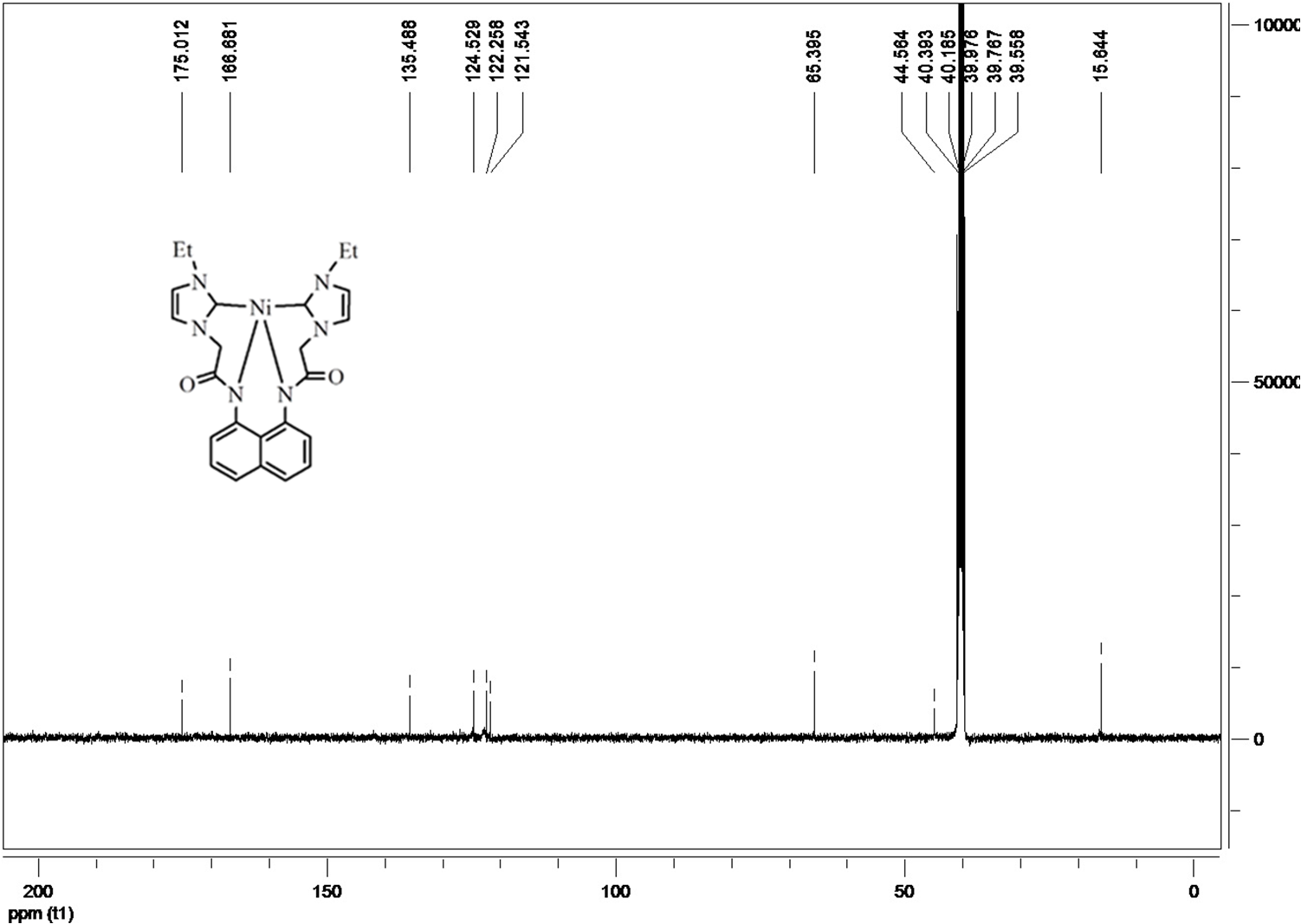


**Figure S18.** The ^13^C NMR (100 MHz, DMSO-*d*_6_) spectra of complex **2**.

**
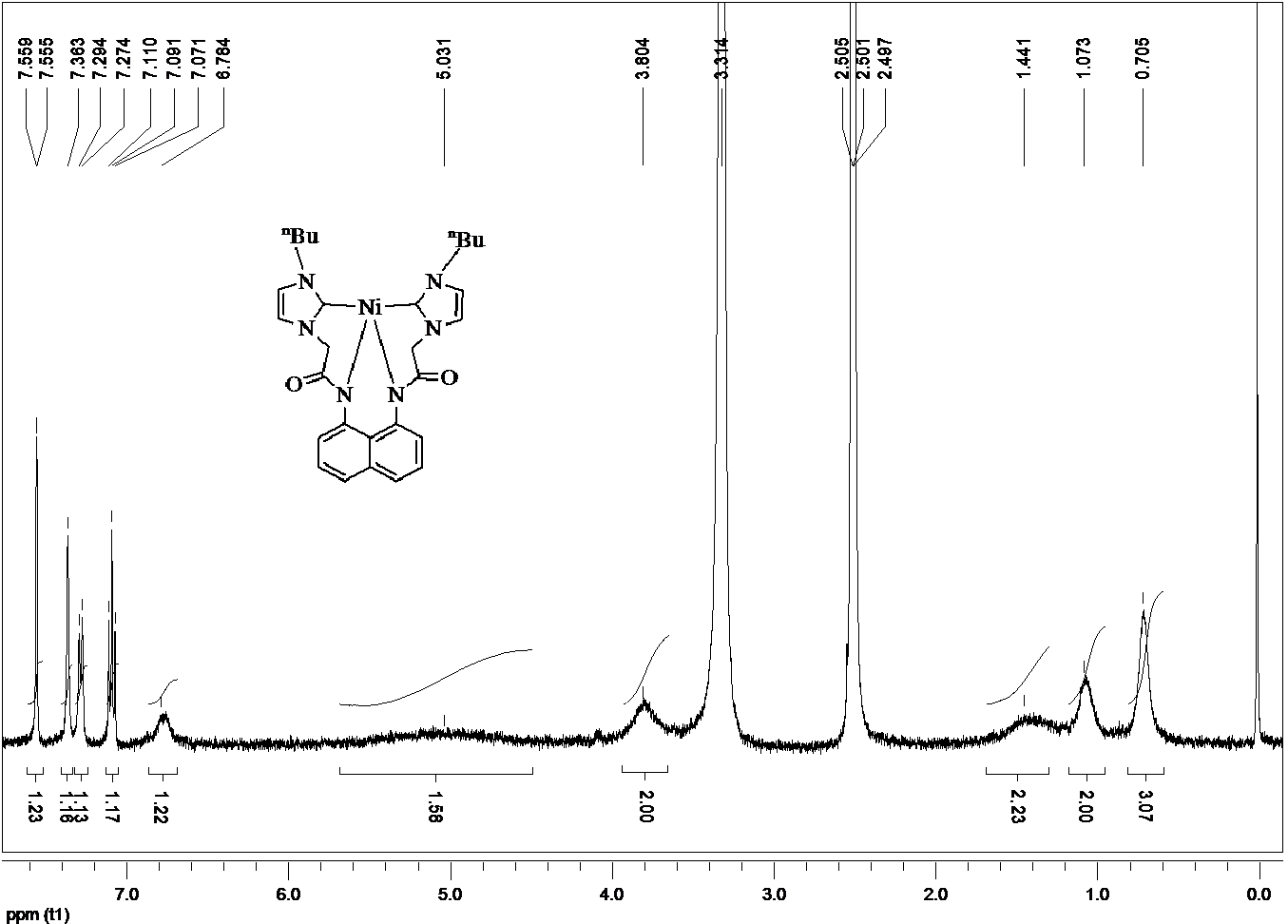
**

**Figure S19.** The ^1^H NMR (400 MHz, DMSO-*d*_6_) spectra of complex **3**.


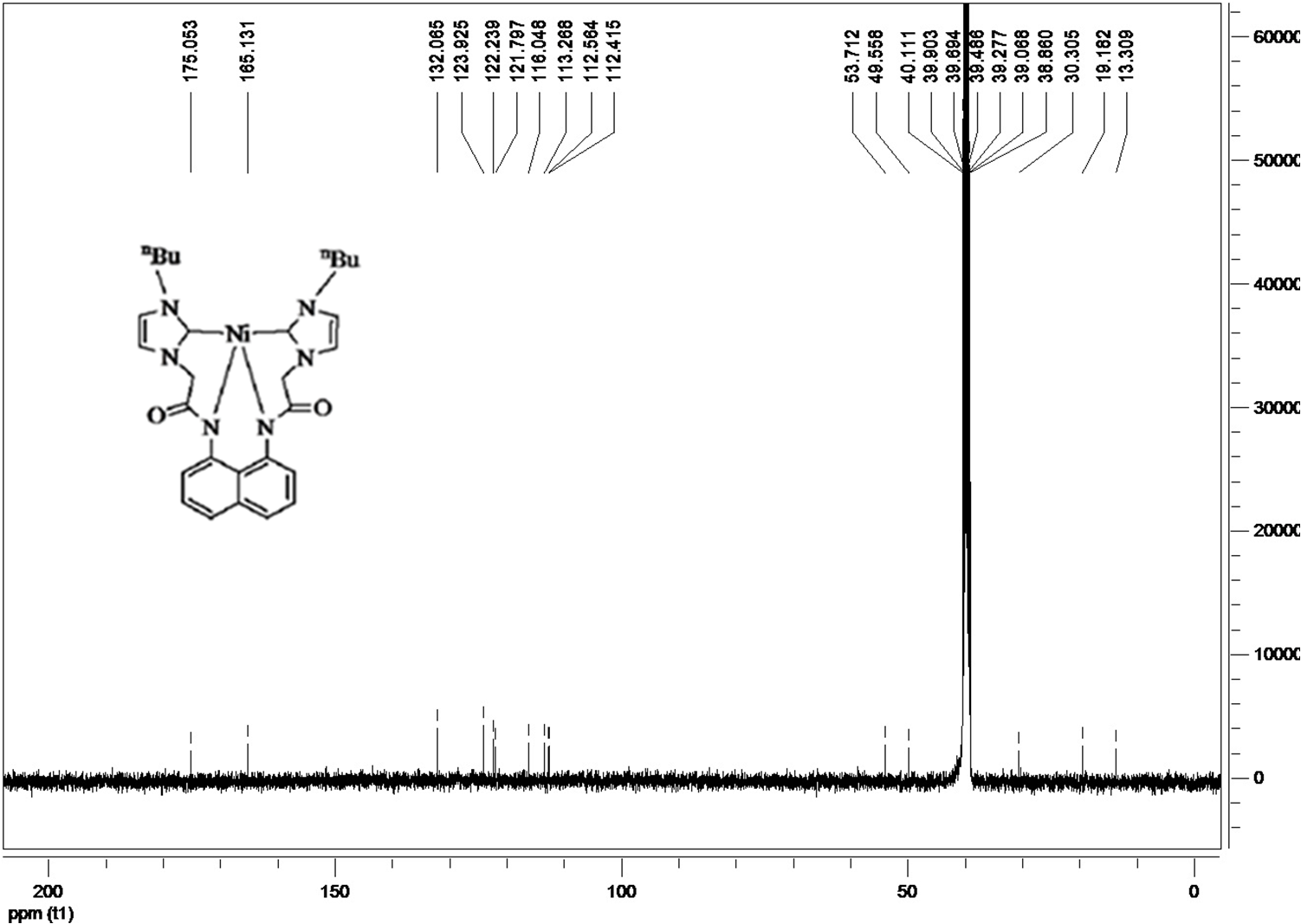


**Figure S20.** The ^13^C NMR (100 MHz, DMSO-*d*_6_) spectra of complex **3**.

**
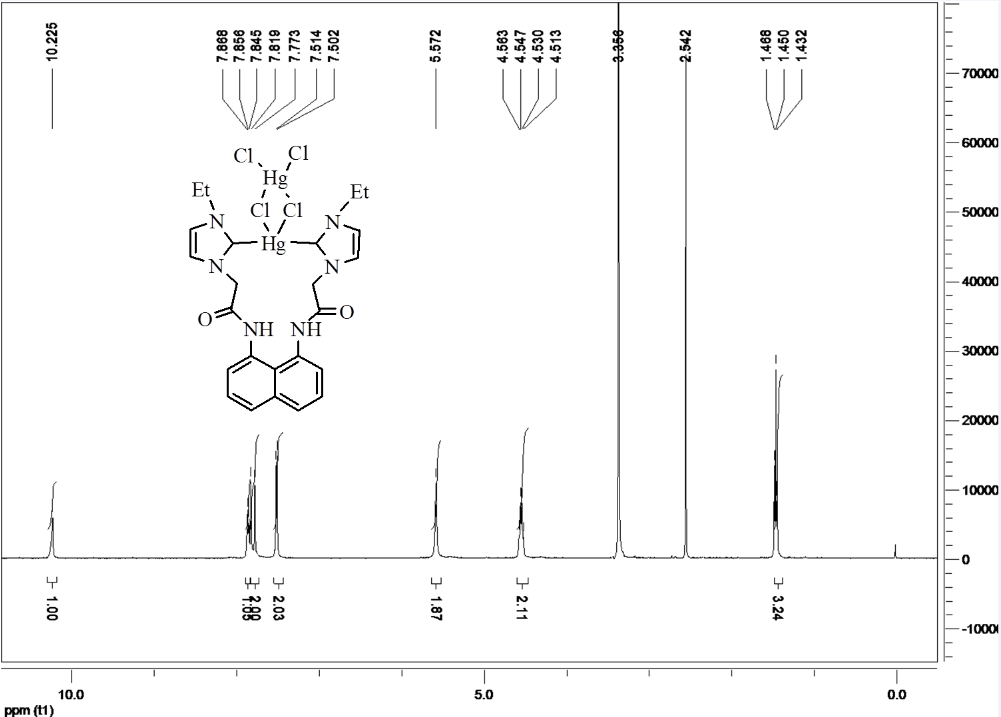
**

**Figure S21.** The ^1^H NMR (400 MHz, DMSO-*d*_6_) spectra of complex **4**.


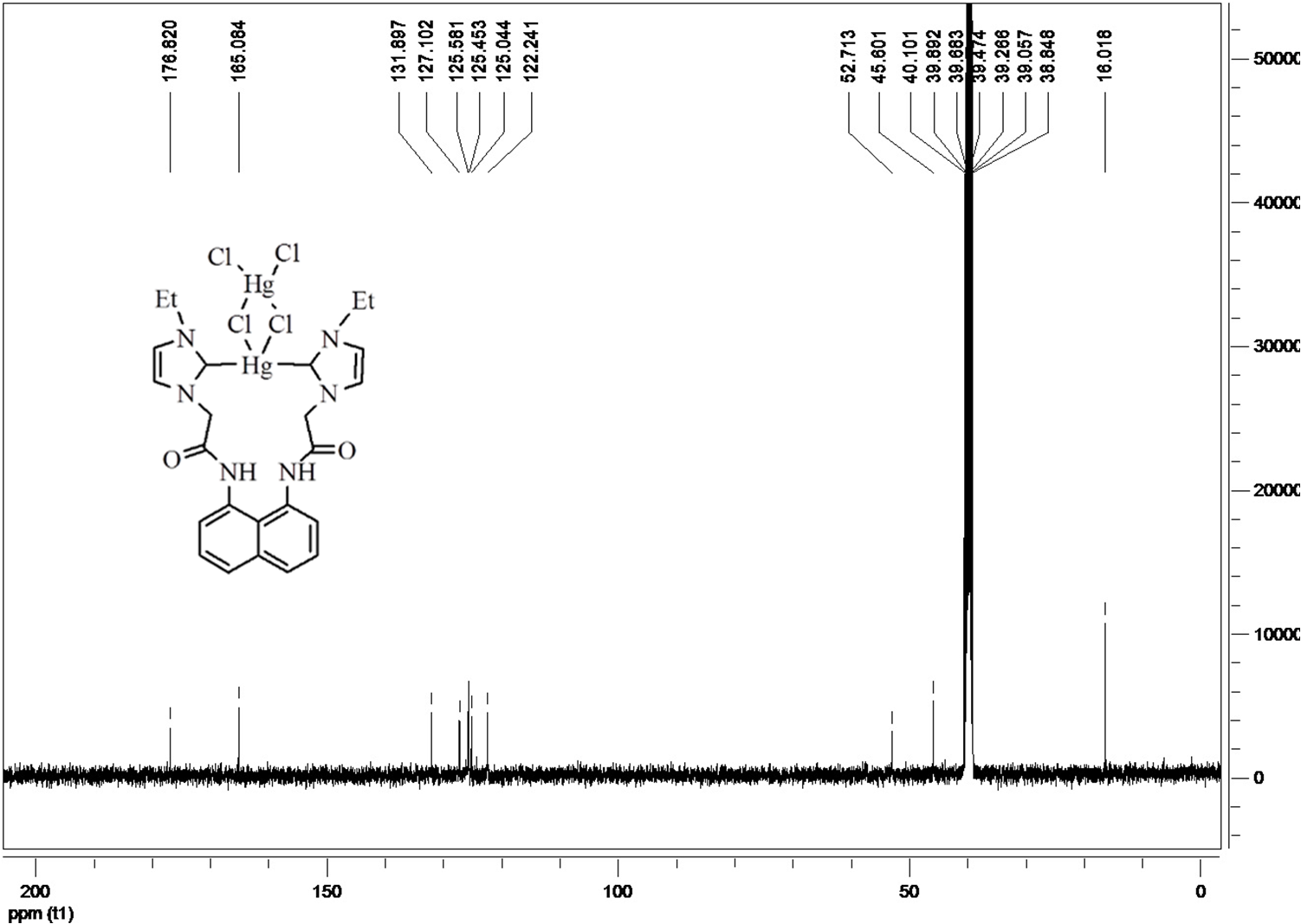


**Figure S22.** The ^13^C NMR (100 MHz, DMSO-*d*_6_) spectra of complex **4**.
